# Projecting future carbon emissions from cement production in developing countries

**DOI:** 10.1038/s41467-023-43660-x

**Published:** 2023-12-11

**Authors:** Danyang Cheng, David M. Reiner, Fan Yang, Can Cui, Jing Meng, Yuli Shan, Yunhui Liu, Shu Tao, Dabo Guan

**Affiliations:** 1 Department of Earth System Sciences, Tsinghua University, Beijing, 100084 China; 2 Judge Business School, University of Cambridge, Cambridge, CB2 1AG United Kingdom; 3 Department of Planning, Aalborg University, 9000 Aalborg, Denmark; 4 Institute of Energy and Process Engineering, ETH Zurich, 8092 Zurich, Switzerland; 5 The Barlett School of Sustainable Construction, University College London, London, WC1E 6BT UK; 6 School of Geography, Earth and Environmental Sciences, University of Birmingham, Birmingham, B15 2TT UK; 7 Research Center for Contemporary Management, School of Economics and Management, Tsinghua University, Beijing, 100084 China; 8 College of Urban and Environmental Sciences, Peking University, Beijing, 100091 China

**Keywords:** Climate-change mitigation, Socioeconomic scenarios, Environmental economics, Climate-change policy, Energy and society

## Abstract

Achieving low-carbon development of the cement industry in the developing countries is fundamental to global emissions abatement, considering the local construction industry’s rapid growth. However, there is currently a lack of systematic and accurate accounting and projection of cement emissions in developing countries, which are characterized with lower basic economic country condition. Here, we provide bottom-up quantifications of emissions from global cement production and reveal a regional shift in the main contributors to global cement CO_2_ emissions. The study further explores cement emissions over 2020-2050 that correspond to different housing and infrastructure conditions and emissions mitigation options for all developing countries except China. We find that cement emissions in developing countries except China will reach 1.4-3.8 Gt in 2050 (depending on different industrialization trajectories), compared to their annual emissions of 0.7 Gt in 2018. The optimal combination of low-carbon measures could contribute to reducing annual emissions by around 65% in 2050 and cumulative emissions by around 48% over 2020-2050. The efficient technological paths towards a low carbon future of cement industry vary among the countries and infrastructure scenarios. Our results are essential to understanding future emissions patterns of the cement industry in the developing countries and can inform policies in the cement sector that contribute to meeting the climate targets set out in the Paris Agreement.

## Abstract (web summary)

The rapid deployment of low-carbon measures is urgently needed to reduce cement emissions as cement CO_2_ emissions from developing countries will almost deplete the remaining cement emissions budget within climate targets.

## Introduction

As one of the largest energy consumers and CO_2_ emitters, the cement industry is a key driver of climate change^1,2^. The sector is currently responsible for 5% to 8% of global anthropogenic CO_2_ emissions every year^3,4^. CO_2_ emissions in the cement industry occur primarily in the production process of clinker – an intermediate product for cement^5,6^ – where CO_2_ is released through both the combustion of fuels for heating and the decomposition of limestone as a chemical process^7^. Within the past few years, China has become the largest contributor of total cement emissions. The cement produced and consumed by China between 2018 and 2020 is more than that produced and consumed by the United States over the entire 20th century^8–10^. However, the cement production in China has decreased since 2015 (see Supplementary Fig. 1).

By 2050, global cement demand is estimated to grow by 12–23% above 2020 levels, driven by the rising global population and urbanization patterns, coupled with infrastructure development needs^11^. Future growth in global cement demand is likely expected to happen in the fast-growing consumers, such as South East Asia and Africa^12,13^. For instance, cement capacity in Ethiopia grew at an annual average rate of 6.8% between 2013 and 2018, and its cement consumption is projected to be 19.97 million tons by the end of 2025, which is more than double its consumption in 2018 (9.09 million tons)^11,14^. To bring the cement sector in line with the Paris Agreement on climate change, its annual emissions need to fall by at least 16 per cent by 2030^15^.

However, reducing emissions from the manufacturing of cement is not straightforward, given the different factors involved, such as kiln type, fuel type, raw materials, and others^16,17^. Roughly half of the greenhouse gas (GHG) emissions from the cement manufacturing process are material-derived, 40% are fuel-derived, electricity accounts for 5% of emissions and transport generates the remaining 5%^3^. There are multiple approaches to reducing carbon emissions, including energy efficiency improvement, fuel substitution, replacing the clinker with cementitious materials, increased production of blended cement, and removing CO_2_ from the flue gas^18–20^. Although the combination of multiple technologies can increase efficiency in emissions reduction, there are challenges and barriers to implementation. So, in the near to medium-term, the cement industry is still incompatible with zero emissions targets, compared to the power sector which could be transformed to zero GHG emissions in a relatively more straight forward manner if strong commitments are made^3^.

Recent studies have made great contributions toward a better understanding of cement emissions and the potential for mitigating those emissions. Many studies analyze emissions reduction pathways for large emitters such as China^21–27^ or have adopted a global perspective^3,11,28,29^. However, there is still a lack of systematic and accurate accounting and projection of cement emissions in other developing countries, especially for the fast-growing consumer markets of South East Asia and Africa.

Here, we provide bottom-up quantifications of emissions from global cement production for the most recent year available (2018) and reveal, according to the age of existing cement plants, a regional shift in the main contributors to global cement CO_2_ emissions. Our global cement CO2 emissions database includes process-related emissions (mainly due to the carbonate decarbonization) and energy-related emissions, however electricity-related emissions are not included. We then develop cement emissions scenarios over 2020-2050 that correspond to different future cement production levels and emissions mitigation options for developing countries except China based on their economic, population and industrial prospects. The scenario analysis is limited to all developing countries^30^ except China, where the vast majority of future growth will occur, yet so far have been understudied. This study aims to help understand future emissions patterns of the cement industry that could inform policies in the cement sector to meet the climate targets set out in the Paris Agreement.

## Results

### Global cement emission patterns

Global emissions from cement production reached 2059 Mt CO_2_ in 2018, where energy- and process-related emissions account for 34% and 66% respectively. Developed countries and economies in transition^30^ contribute 13% of emissions from global cement production despite their high level of wealth and large population because these countries have now largely concluded large-scale construction and as a result have a lower demand for cement than in their earlier development stages. By contrast, China, and other developing countries are the main contributors, responsible for 52% and 35% of emissions from global cement production, with 1319 and 1163 cement plants, respectively (see the composition structure of global cement capacity in Supplementary Table 1). The largest emitters are in Asia. China led the world with a total of 1073 Mt CO_2_, followed by India, Vietnam, and Indonesia with 159, 48, and 44 Mt CO_2_ respectively. Unsurprisingly, the ranking of main contributors to emissions from global cement production varies over time, depending on economic growth, industrialization, and urbanization (see examples in Supplementary Fig. 1).

**Fig. 1:**
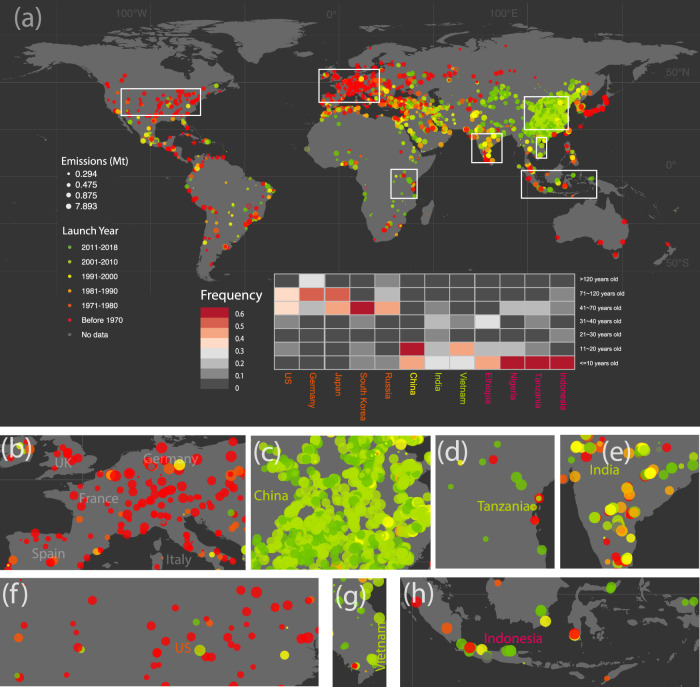
Global cement CO_2_ emissions. **a** Spatial distribution and age structure of global cement CO_2_ emissions. Each point represents a cement plant, with its color referring to the launch year, and the size of the circle representing CO_2_ emissions in 2018. The heat map presents the ratio of emissions by age of cement plant and country. Spatial distribution of hotspot of cement CO_2_ emissions in seven selected regions or countries: Europe (**b**), China (**c**), Tanzania (**d**), India (**e**), United States (**f**), Vietnam (**g**) and Indonesia (**h**).

Here we group countries into 4 sets according to the age of existing cement plants (see Supplementary Methods for detailed classification method) and illustrate the dynamic patterns of cement production by group. In Group 1 countries, which consists of developed countries and some economies in transition (e.g., United States, Germany, South Korea, Japan, Russia), a large number of cement plants were built from around 1900 onward with very few new plants built after 1990 (see Fig. 1). This pattern shows that the extent to which the basic infrastructure in Group 1 countries had been mostly deployed by 1990. For instance, the US had achieved a high level of both total and per capita cement production before 1970 (see Supplementary Figs. 1 and 2). As the growth pattern of cement production explains the variation of cement emissions, it follows that cement emissions in Group 1 countries increased rapidly before 1970, then grew steadily or stayed constant, with some countries showing emissions reduction after 2000 (e.g., Netherlands)^31^.

In Group 2 countries, the largest cement producer, China, started most large-scale construction of cement plants around the year 2000 and experienced rapid growth in production from around that time. More specifically, 92% of existing cement plants in China were built between 2000 and 2018. As a result of the rapid growth in cement production, China’s total annual CO_2_ emissions and per capita CO_2_ emissions from cement ranked highest in the world, reaching 1073 Mt and 768.8 kg in 2018, respectively, driven in part by the doubling of urban per capita floor area from 20.3 sqm in 2000 to 39 sqm in 2018 according to the *National Bureau of Statistics*^32^, reaching a level now comparable to many European countries. Similar to the countries in Group 1, after a rapid growth phase, China’s cement production peaked in 2014 and has been declining since then (see Supplementary Fig. 1).

Looking at Group 3 countries (India, Vietnam, etc.), cement plants have a similar age to those in Group 2; here countries started large-scale construction of cement plants and experienced rapid growth of cement production (and emissions) around the year 2000. However, the growth rate varies dramatically between the two groups. More specifically, cement production in India grew at an average annual rate of 6.6% during 2000-2015, while in China it grew at an average rate of 11.7% over the same period. The slower growth rate meant delayed infrastructure development. Over 1970-2018, per capita cumulative cement production in India amounted to only 4257.8 kg, and it was coupled with a much smaller floor area of 10 sqm per capita (in 2014) than in China.

Finally, Group 4 countries are at the lowest levels of housing and infrastructure development. Some Asian and African countries, such as Indonesia, Myanmar, Egypt, and Tanzania, began large-scale cement manufacturing only after 2010. For instance, per capita cement production over 1970-2018 was only 2370 kg cumulatively in Tanzania, approximately 6 times less than the level of the US from Group 1, even two times less than India from Group 3. However, Group 3 and Group 4 countries have growing populations and are experiencing rapid economic development and a demand for a better quality of life, which means an increasing demand for housing and infrastructure and will lead to an increase in cement production as well as cement emissions.

In addition to the heterogeneous patterns of cement emissions, there are also sharp differences in emission intensity across different countries. Among the major cement producer countries shown in Supplementary Fig. 3, the lowest total emission intensity globally was actually in China with 0.46-ton CO_2_ per ton of cement. Despite its reliance on coal, which has the highest CO_2_ emission factor among various fuels, China has a low clinker-to-cement ratio as well as the most energy-efficient kilns making it a leader in the energy efficiency of the global cement industry. However, it is noticed that China is now starting to use fewer SCMs due to the concerns about the cement quality, and may witness higher clinker-to-cement ratio in the near future^16,31^. Moreover, China has invested in construction in and has given technical support directly to, some African countries thereby helping them achieve relatively low emission intensity production (e.g., Tanzania)^33–35^. For many other countries, however, there still exists great potential to improve material efficiency. Russia’s emission intensity exceeds 0.60-ton CO_2_ per ton of cement, compared to a global average emission intensity of 0.56-ton CO_2_ per ton of cement. To explore the driving factors, on the one hand, the Russia’s clinker factor to cement is still very high with 87%, compared to the global average level with 65%^11^. There is a large proportion of Portland cement produced without mineral additives in Russia (62.5 per cent of total production), according to the *International Cement Review*. On the other hand, Russia’s high emissions intensity could be attributed to the low thermal efficiency. Over two thirds of current cement capacity in Russia are sourced from the old plants that were built before 1975. And 60% of these old plants are installed with wet kilns which require higher energy input than the dry kilns most commonly used now.

### Projection of future emissions growth in developing countries

Most developing countries expect rising population and infrastructure development needs over the next decades^11^, and as a result, they are projected to experience rapid growth in their domestic cement production. We set four scenarios for cement production over 2020-2050, which correspond to different demands to expand the built environment following economic and population growth (*Tier 1 cement production levels*). The scenario analysis is limited to all developing countries^30^ except China. When we refer to developing countries in the following, China is not included. First, the ‘business as usual (BAU)’ scenario presents a trajectory of infrastructure expansion that follows the historical trend estimated by the International Energy Agency^36,37^; second, the ‘Global Average’ scenario assumes that building on BAU, some countries will accelerate the expansion of construction to catch up with those in the same group that are more advanced in housing condition so that in 2050 all countries will reach the average level of global countries’ housing conditions in 2020; third, the ‘China Level’ scenario projects that, in 2050, all countries will reach the housing condition of China in 2020; fourth, the most ambitious ‘Developed Average’ scenario projects that, by 2050, all countries will reach the level of housing condition equal to the average level in developed countries in 2020. Full scenario settings are provided in Supplementary Methods.

**Fig. 2:**
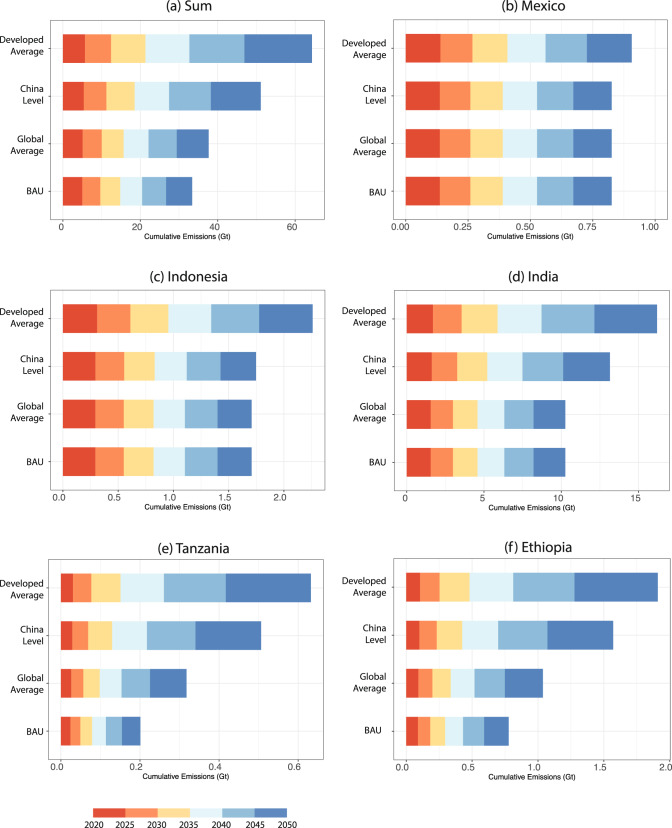
Projected future cement emissions. **a** Sum of projected cement emissions for all developing countries except China. Projected cement emissions in the following five countries: Mexico (**b**), Indonesia (**c**), India (**d**), Tanzania (**e**) and Ethiopia (**f**) (see description of countries in Note S1). Each plot contains four scenarios presenting the estimated cement emissions based on different paths to expand the built environment: BAU (business as usual), Global Average, China Level and Developed Average. The length of bars corresponds to cumulative emissions (Gt) under different scenarios, with the colors indicating the time periods.

Figure 2 shows the projected emissions under different cement production scenarios across developing countries as well as for selected countries. Under the BAU scenario, developing countries are projected to experience rapid growth in annual cement CO_2_ emissions, rising from 793 Mt in 2020 to 1395Mt in 2050, a level as high as the annual cement emissions of China in 2050 according to the IEA^11^ (Supplementary Fig. 5). Given rising annual emissions, the level of cumulative cement CO_2_ emissions in developing countries is projected to reach 33407 Mt over 2020-2050 under the BAU scenario (Fig. 2a). Compared to the BAU scenario, the increasing demand for infrastructure will drive the growth of cement production as well as cement CO_2_ emissions. The highest infrastructure demand under the Developed Average scenario will result in additional 30924 Mt cumulative cement CO_2_ emissions during the period 2020-2050, whereas under the other two more moderate scenarios (Global Average and China Level scenario) cumulative CO_2_ emissions will increase by 4246 Mt and 17712 Mt, respectively. At the same time, average cement CO_2_ emissions per capita in the developing countries in 2050 show a dramatic shift from 0.30 t in the BAU scenario to 0.79 t in the Developed Average scenario, which is comparable to US levels in 2018 (0.77 t CO_2_ /cap). Furthermore, sensitivity analysis results show that the 10^th^ and 90^th^ percentile of the cumulative emissions over 2020-2050 under the Developed Average scenario are 39840 Mt and 95280 Mt respectively (see Supplementary Figs. 9, 10, Supplementary Tables 14, 15). This implies that, despite the uncertainties, the Developed Average scenario would result in substantial additional cement emissions in most cases. Our results quantify the growth of cement emissions under ambitious but still possible socio-economic development pathways and point out that the potential growth in the demand for cement in developing countries might have been substantially underestimated by the past studies.

Looking at different regions, there is no dramatic difference in the level of emissions under the four cement production scenarios for some wealthier Asian and Latin American countries, like Mexico in Fig. 2b. This is because, given the higher starting point and the faster pace at which infrastructure develops compared to other countries, Mexico is projected to easily reach the infrastructure conditions of developed countries in the BAU scenario. By contrast, some growing Asian and Latin American countries like Indonesia and Peru are projected to achieve the same infrastructure conditions as China but remain behind the average level of developed countries, whereas other Asian and Latin American countries like India and Bolivia will only reach the average level of developing countries in the BAU scenario, as shown in Fig. 2c, d. Regardless of the expected cement production, Asia (excluding China) will remain the largest emitter among developing countries. Finally, in the most cases of African countries, under each scenario representing a higher demand for infrastructure than the BAU scenario, the countries will experience dramatic emissions growth because of their currently poor infrastructure condition and large, growing populations. For instance, cumulative emissions over 2020-2050 in Tanzania are projected to grow from 201 Mt in the BAU scenario to 632 Mt in the Developed Average scenario, and more than half of cumulative emissions will be emitted during 2040–2050. Given the heterogeneity of projected emission patterns, underlying country-specific factors, such as historical patterns in infrastructure and population growth, need to be considered in designing any specific emissions reduction plan.

### The potential of emissions reduction in the cement industry

In the context of meeting the different levels of cement production for future construction (*Tier 1*), we further investigate the mitigation potentials of cement emissions in developing countries (except China) under the different combinations of low carbon measures (*Tier 2 emissions mitigation options*). We calculate the emissions mitigation potential of the four options commonly discussed in the cement industry^29^. The options considered are thermal efficiency improvement (K), waste fuels (W), carbon capture and storage (C) and supplementary cementitious materials (S). Each option has a lower and upper limit, for instance, C_1_ refers to no adoption of carbon capture and storage (business, as usual, BAU) and C_2_ refers to the adoption of carbon capture and storage with a capture efficiency of 95% (see detailed scenario descriptions in Methods and Supplementary Methods). The extremely low carbon (LC) emission scenario (K_2_W_2_S_2_C_2_) assumes that the four low carbon measures are fully implemented, while the baseline emission scenario has no low carbon measures.

**Fig. 3:**
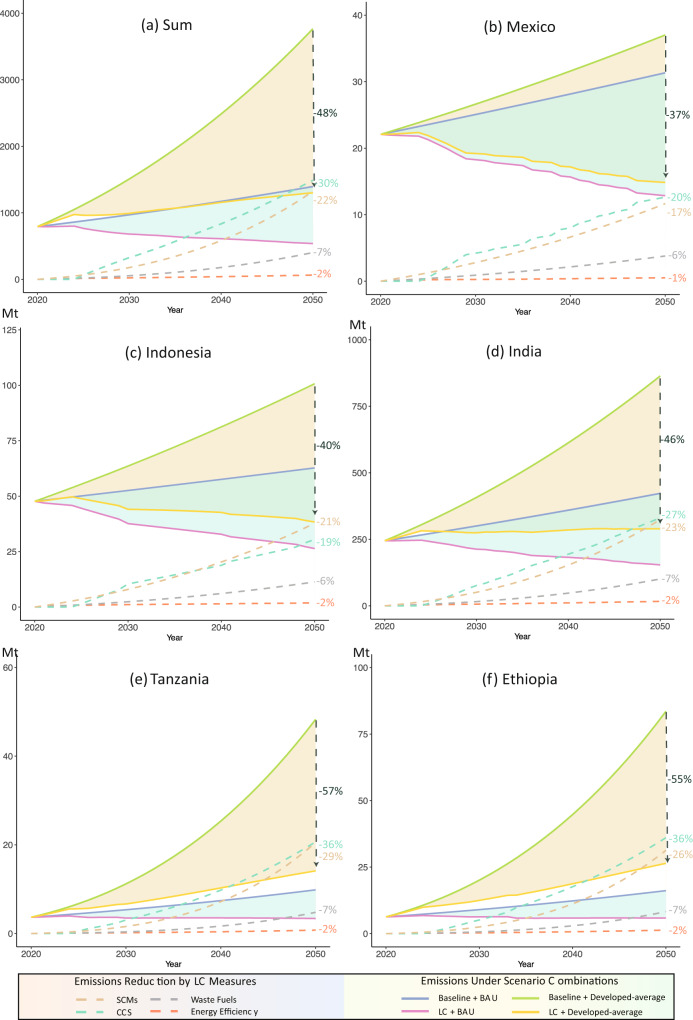
Cement emissions reduction potential. **a**, total cement emissions from all developing countries except China under different cement production scenario sets (BAU and Developed Average) and emission mitigation scenario sets (Baseline and Extremely Low Carbon). b-f, cement emissions of the following selected countries under the same scenario combinations: Mexico (**b**), Indonesia (**c**), India (**d**), Tanzania (**e**) and Ethiopia (**f**). The solid line indicates the projected annual cement emissions over 2020–2050. The dark color refers to the Baseline Emission and light color refers to the Extremely Low Carbon (LC) Emission. Orange and green represent the BAU and Developed Average scenarios, respectively. The dashed line indicates the cement emissions reduction of four low carbon measures, carbon capture and storage (CCS) technologies, supplementary cementitious materials (SCMs), Waste Fuels and Energy Efficiency. The percentages listed on the right-hand axis indicate the cumulative emissions reduction of different low carbon measures relative to the Baseline, meeting the Developed Average level of cement production, and the percentages on the right of the dark green dashed lines correspond to the cumulative emissions reduction under Extremely LC scenario.

Figure 3 gives comparisons between the extremely LC emissions and baseline emissions for developing countries over 2020-2050, under BAU and Developed Average cement production scenarios respectively. The results show that the combination of low-carbon measures contributes to reducing developing countries’ cement emissions substantially. Under the BAU scenario, cumulative cement emissions from developing countries during 2020-2050 are reduced from 33407 Mt to 20402 Mt by aggressively adopting the four low-carbon measures discussed here (Fig. 3a). The resulting reduction of 13 Gt exceeds the cumulative cement emissions in India in the BAU scenario (10 Gt) over the same period. The mitigation effects of the extremely LC scenario will be larger with growing cement production as a result of increasing demand for infrastructure. More specifically, the rate of reduction in cumulative emissions under the extremely low carbon measures will go from 38.9% under the BAU scenario to 48.0% under the Developed Average scenario. This is because the substantial growth of cement production expected in the latter scenario requires newly installed capacity, which is more likely to be less carbon-intensive as investors opt for low carbon technologies.

Among the four low-carbon options considered, the potential mitigation outcomes of the combined measures vary by country and infrastructure demand due to differences in development status and response capacity. For example, for CCS technology, the reduction potential of cumulative emissions over 2020–2050 ranges between 13.3% and 36.4%, bounded by the case of Indonesia under the BAU cement production scenario at the lower end and Ethiopia under developed-average cement production scenario at the upper end (Fig. 3 and Supplementary Fig. 6). This range can be mainly explained by the fact that the percentage of new installed capacity varies between different countries and infrastructure target, because CCS technology is more feasible and applicable in the newly built cement plants^38^. For example, in Ethiopia, emission reductions attributed to CCS grows from 24.5% to 36.4%, as the percentage of new installed capacity increases from 73.5% under the BAU cement production scenario to 94.9% in the Developed Average cement production scenario. For the countries under the Developed Average scenario where a high percentage of new capacity would be installed to satisfy the growing infrastructure needs, CCS is the most efficient measure to mitigate emissions from cement. Conversely, in scenarios with limited newly built capacity, SCMs would be a more promising option considering its high reduction potential on existing stock and low economic cost^39^. For example, in India under BAU cement production scenario, the emission reduction ratio from SCMs (20.5%), is higher than that of CCS (19.9%). Additionally, the emission reduction potential of SCMs would be more appealing in countries with the current high levels of clinker-to-cement ratio.

Furthermore, the emission reduction potentials of low carbon measures vary by each cement plant. On the one hand, the differences of production technologies among cement plants result in the heterogeneity of emission mitigation potentials. The cumulative emission reduction ratio of kiln retrofitting over 2020–2050 is under 2% for the cement plants with dry kiln, while that ratio would exceed 6% for the cement plants with wet or draft kilns. On the other hand, the early retirement would reduce the cumulative emissions mitigation potential of low carbon measures for cement plants. Under the BAU cement production scenario, the cumulative emission reduction ratio of adopting SCMs would be over 12% for the well-working cement plants, while that ratio would decrease to 6% for the cement plants that retire before 2030. Every plant deserves a low carbon pathway in terms of their own age, kiln type, etc. Despite that the kiln energy efficiency improvement contributes the least to the country-level cement emission mitigation, it could be a preferrable choice for some particular cement plants- the young cement plants with large cement capacity and low-efficiency kilns.

Based on the efficient low-carbon paths and strategies, it is possible for most Asian and Latin American countries to achieve negative or negligible growth in annual cement emissions before 2050. For instance, under an extremely LC scenario with the level of cement production under the Developed Average, the annual rate of increase in cement carbon emissions over 2020-2050 in India is 0.6%, compared to the rate of 4.3% without low carbon measures. However, annual cement emissions in African countries will continue growing fast and it will not be possible to completely offset the additional emissions brought by the highest demand for infrastructure, even if all low-carbon measures are adopted. For instance, in Tanzania, although the extremely LC scenario offers a great emissions reduction potential at around 57.3%, annual extremely LC cement emissions under the highest cement production scenario continuously grow from 3.7 Mt in 2020 to 14.1 Mt in 2050 which is substantially higher than 9.8 Mt under the BAU scenario without low carbon technologies. This outcome is explained by the rapid growth of cement demand from infrastructure systems in African countries. In summary, even the extremely LC emissions scenario that follows the cement technology roadmap proposed by IEA^11^ cannot avoid the rapid growth of cement emissions or offset the great volumes of additional emissions created by the rapid growth rates at which construction happens in many African countries.

## Discussion

This study makes three contributions to the research field of global cement emissions accounting. First, the global plant-level cement database compiled in this study enables a more accurate emission accounting as well as a closer to real life emission mitigation assessment for the developing countries. Second, this study advances the methodology regarding the future cement emissions projection through quantifying the cement emissions driven by various socio-economic scenarios. Third, our results show that the mitigation outcome of low carbon measures varies among different countries and infrastructure scenarios. Our results help decision-makers from developing countries to formulate scientific policies or pathways in cement emission mitigation based on local circumstances.

Our research reveals that driven by the need to expand infrastructure, developing countries are likely to experience rapid growth in CO_2_ emissions from the cement industry over 2020–2050. Cumulative cement emissions of all developing countries (except China) over the next three decades are projected to exceed 33 Gt under the BAU scenario (see Supplementary Fig. 7). Under more aggressive growth assumptions, cumulative emissions may increase by an additional 31 Gt. The cement CO_2_ emissions from these countries will almost deplete the remaining cement emissions budget for the 2°C climate target (41 Gt CO_2_ emissions) and makes it impossible to achieve the 1.5 °C climate target (33 Gt CO_2_ emissions). The rapid deployment of low-carbon measures is urgently needed to reduce cement emissions and not exceed the carbon budget. The full implementation of four distinct low-carbon measures in the cement sector (extremely LC scenario) could contribute to reducing cumulative emissions by 48% during 2020-2050 relative to the baseline in the case of developing countries. According to the level of cement production under the BAU scenario in combination with the extremely LC scenario, developing countries (except China) should be allowed to emit at least 20 Gt over 2020-2050. It would require the rest of the world to reduce their cement emissions at an annual rate of 9% or 5%, respectively, to meet the global target of 1.5 or 2 °C (see Supplementary Fig. 8). If instead, we consider the level of cement production under the Developed Average scenario, the emissions space for these countries grows to 33 Gt, whereas the rest of the world should reduce emissions by 18% annually in order to reach the 2 °C target. Hence, low carbon measures should be widely adopted across both the developed and developing countries, in order to ensure that emissions from the cement industry remain in line with a 1.5 or 2 °C target over the following decades.

In order to mitigate emissions efficiently, each developing country needs to formulate policies or pathways based on local conditions. Our results demonstrate that the best path towards low-carbon cement future varies among countries and depends on infrastructure conditions. For the scenarios where rapid capacity expansion is needed to satisfy the growing infrastructure demand, using CCS makes the most important contribution to emission mitgation. For example, in the developed-average cement production scenario it will reduce cumulative emissions of developing countries by 30% on average during 2020–2050. While in cases where capacity expansion is limited, using SCMs is a promising solution to reduce cement emissions with high efficiency and low economic cost. Great efforts should be channeled towards greening the cement industry in African countries, which are characterized by continuous fast-growing cement emissions driven by the high infrastructure demand.

Since the extremely low carbon emissions scenario cannot avoid the rapid growth of cement emissions in developing countries, it is essential for the developed countries to strengthen low carbon measures in cement production as well as other carbon-intensive industries, in order to give the necessary emissions quota to developing countries for infrastructure system upgrade. Six countries – the Netherlands, Sweden, Germany, France, the United Kingdom and United States – are in the vanguard of adopting low carbon concrete and construction rules and green procurement policies examined by the EU Green Public Procurement Program, which could be a blueprint for other developed countries. Moreover, higher-income countries should provide considerable economic support and facilitate technology transfer to the developing countries that face technological barriers to reduce emissions^40–42^. For instance, Kenya and the UK have a long tradition of cooperation and collaboration through research and scientific partnerships and technology transfer for sustainable construction, including a research symposium on ‘Healthy Cities, Housing and Sustainable Infrastructure’ and a £15 million UK-funded research project on housing in Kenya aiming to create energy-efficienct construction materials^43^.

## Methods

### Construction of Global Cement Emissions Database

The comprehensive database of global cement plants includes 3094 cement plants, of which 3020 are integrated plants and 74 are clinker plants. The database provides information on plant name, site location, operator, host country, cement capacity, type of works (integrated or clinker) for all cement plants, and year of commissioning and type of kiln (dry, semi-dry, semi-wet, wet, shaft and new dry kiln) for the majority of the plants (see Supplementary Table 1 for the composition structure). New dry kilns refer to the advanced new dry rotary kilns with suspension pre-heaters or pre-calciners that are widely adopted in China. Compared to the best-available list of global cement production sites^3^, our study curates a more extensive and more detailed database using the latest available data (from 2018), by adding another 700 cement plants and including plant-specific information on kiln type and year of commissioning. The detailed process parameters for cement plants in our database allow a more accurate estimation of current and hence future emissions from global cement production.

Our global cement CO_2_ emissions database includes all process- and energy-related emissions, however electricity-related emissions are not included. The process-related carbon emissions represent the CO_2_ emitted during the calcination of raw meal, in which the limestone is heated to lime and carbon dioxide. In this study, the process-related carbon emissions are estimated using Eq. (1)^44^:

1 $${E}_{{process},a,t}={{AD}}_{{clk},a,t} * {{EF}}_{{calcination},c}$$

where ${{AD}}_{{clk},a,y}$ refers to the clinker production of the plant *a* in year *t*; ${{EF}}_{{calcination},c}$ represents the country-level emissions factor for the clinker production during the calcination of raw meal, that is, the CO_2_ emitted per unit production of clinker.

The direct energy-related CO2 emissions are estimated as following:

2 $${E}_{{combustion},a,t}={{AD}}_{{clk},a} * {{EI}}_{k} * \sum \left({S}_{i,c} * {{EF}}_{{fuel},i,c}\right)$$

Where ${{EI}}_{k}$ denotes energy intensity (J/kg clinker) of the kiln type of *k*; ${S}_{i,c}$, and ${{EF}}_{{fuel},i,c}$ represent the share, and emission factor of the i_th_ type of fuel in the country or region *c* where the plant *a* is located; i represents the different type of fossil fuels used to supply energy, including oil, coal and natural gas. Biomass fuels are excluded in the energy-related emissions accounting, which are supposed to be accounted in the (Land Use, Land-Use Change and Forestry) LULUCF sector stated by the United Nations Framework Convention on Climate Change (UNFCCC)^45^. Note that energy intensity of any particular kiln is determined by number of preheaters, burning technologies and other technological characteristics, and affected by its clinker composition. Nevertheless, due to limited data availability, we adopt a global average energy intensity for each kiln type as a proxy, similar to past studies^3^.

### Projection of future cement demands and emissions

#### Scenario description

To evaluate future CO_2_ emissions from cement production in emerging economies, we propose two scenario sets (tier 1 and tier 2) that correspond to different levels of cement production and emissions mitigation options respectively. The scenario analysis is limited to all developing countries except for China (see the list in Supplementary Table 5, which is published by the *World Economic Situation and Prospects* 2020^30^). Developing countries are characterized with lower basic economic country condition. Complete scenario descriptions are provided in Supplementary Methods.

More specifically, Tier 1 scenarios present the estimated cement production based on different levels of cement demand required to expand the built environment. It is assumed that all domestic cement demand will be always met by local production^46^. We define four scenarios for the projection of cement demand, which correspond to varying degrees of improvement in the infrastructure systems: BAU, Global Average scenario, China Level scenario and Developed Average scenario. The four scenarios are characterized by different growth rates in per capita floor area.

##### BAU

anticipates that infrastructure will grow according to the speed of GDP per capita by country under SSP2^47^. This trend broadly agrees with the growth rate for floor area published by the IEA^36,37^ (see Supplementary Fig. 4).

##### Global Average scenario

building on the BAU, all countries which do not currently meet a level of 29 m^2^ floor area per person will linearly accelerate construction to achieve this target in 2050. In 2050, all countries will reach the same level of housing conditions that was achieved globally in 2020.

##### China Level scenario

similarly, all countries that do not currently meet the level of 40 m^2^ floor area per person will accelerate construction to achieve this target in 2050. In 2050, all countries will reach the level of housing conditions that China achieved in 2020.

##### Developed Average scenario

finally, extending our ambition even further, countries that do not currently meet the level of 47 m^2^ floor area per person will accelerate construction to achieve this target by 2050. In 2050, all countries will reach the average level of housing conditions that developed countries achieved in 2020.

Tier 2 scenarios present the possible mitigation measures, which consist of thermal efficiency improvement (K), waste fuels (W), carbon capture and storage (C) and supplementary cementitious materials (S). Each option has a lower and upper limit, for instance, C_1_ is in line with the current situation and C_2_ refers to the adoption of carbon capture and storage. We treat K, W, S, C as individual variables in the model, and therefore we end up with 16 scenario combinations in the tier 2 scenario set. The baseline emission refers to the scenario of the combination of measures K_1_W_1_S_1_C_1_, and the extremely low carbon refers to the mitigation scenario K_2_W_2_S_2_C_2_. In the baseline scenario, all parameters regarding energy structure, clinker factor, thermal intensity, etc., are in line with the current situation. The specific measures and technical parameters adopted in the scenarios are described below. More details about the scenarios can be found in Supplementary Methods.

##### Kiln Energy Efficiency

This scenario considers the mitigation effects of improving kilns’ energy efficiency. In **K**_**2**_, existing cement plants with wet, semi-wet, semi-dry and shaft kilns will be retrofitted to dry kilns before 2030 based on a linear decline from 2021 and all newly-built cement plants will be retrofitted with dry kilns. All dry kilns would be implemented with the pre-decomposition kiln to increase the efficiency. Comparatively, in **K**_**1**_, existing cement plants will be in line with the current situation and all newly-built cement plants will be built with dry kilns. This study assumes no improvement in energy efficiency for kilns over time. The current growth of the most widely adopted dry or new dry kiln efficiency is very slow as it is close to the saturation levels (see the evidence of global time-series energy consumption intensity data from *WBCSD* in Supplementary Table 17).

##### Waste Fuels

In **W**_**2**_, the share of alternative fuels in the energy mix is assumed to linearly increase to 30% by 2050 from the current level, compared to **W**_**1**_, where alternative fuels are in line with the current situation.

##### Supplementary cementitious materials

Clinker is the main ingredient in cement, and the amount used is directly proportional to the CO2 emissions generated in cement manufacturing, due to the combustion of fuels and the decomposition of limestone in the clinker production process. The decreasing clinker-to-cement ratio will be needed to get on track with the low-carbon cement roadmap. In the **S**_**2**_ scenario, we assume a clinker-to-cement ratio of 0.50 in 2050, which is achieved by a linear decline from the existing clinker-to-cement ratio for each country from 2021. By contrast in **S**_**1**_, we assume no change in the country-specific clinker-to-cement ratio.

##### Carbon capture and storage

In **C**_**2,**_ the efficiency of carbon capture is set at 95% before 2050, and 50% of future new cement capacity and 10% of existing cement capacity will be implemented with CCS technology, whereas in **C**_**1**_ no CCS is deployed.

#### Cement demand projection framework

The framework for projecting cement demand includes three sub systems: (i) residential buildings; (ii) non-residential buildings and (iii) civil engineering. We employ the model established by Hong^48^ to estimate the cement demand for residential buildings. This model takes floor area as the proxy and is essentially grounded on a stock-driven model. The stock-driven model was introduced by Müller^49^ in 2006 as an alternative method for simultaneously forecasting resource demand, which is now widely used by the material flow analysis community^50–53^. See details of the framework in Supplementary Methods and Supplementary Fig. 12- Supplementary Fig. 13.

The mathematical equations used to estimate the total floor area of the residential building in country *c* in year *t* are described below.

3 $${{SR}}_{c,t}={P}_{c,t} * {{ar}}_{c,t}$$

where ${{SR}}_{c,t}$ is the total floor area of the residential building stock, ${P}_{c,t}$ is the population in country *c* in year *t* and ${{ar}}_{c,t}$ is the per capita floor area of residential buildings.

4 $${N}_{c,t,t-1}={{SR}}_{c,t}-{{SR}}_{c,t-1}+{D}_{c,t,t-1}$$

where ${N}_{i,t,t-1}$ is the newly built residential building in country *c* in year *t*, and ${D}_{c,t,t-1}$ is the demolished residential building because buildings will, of course, be dismantled after their service lifetime. The country-specific data for demolished residential buildings is estimated by multiplying the demolition rate and country-specific total residential building floor area. Due to data availability constraints, we adopt a demolition rate of 0.5% for all developing countries^54–56^, except for India where we use a rate of 1.43%^54,57^. According to our sensitivity analysis (see Supplementary Methods), the uncertainty in demolition rate does not have a significant effect on total cement demand globally.

Total cement demand for residential buildings (${{CR}}_{c,t}$) in country *c* in year *t* can be expressed as Eq. (5), multiplying the cement intensity of residential buildings ${{CIR}}_{c,t}$ and newly built residential building floor area. In other words, the cement demand is directly decided by the newly built residential floor area and cement intensity. Since the cement intensity is constant over time in most cases (see Supplementary Table 8), each country’s annual cement demand grows proportionally to its local newly built residential floor area.

5 $${{CR}}_{c,t}={N}_{c,t,t-1} * {{CIR}}_{c,t}$$

However, it is difficult to account for the total of non-residential buildings as well as infrastructure stock due to data availability. Hence, we simplifies the accounting method by adopting a region-specific ratio between residential buildings and non-residential buildings and civil engineering infrastructures, shown as Eq. (6).

6 $${{CO}}_{c,t}={{CR}}_{c,t} * {{tr}}_{c,t}$$

where ${{CO}}_{c,t}$ is the cement demand of the non-residential building plus civil engineering in country/region c in year t, ${{tr}}_{c,t}$ is the transition rate that represents the difference of the cement demand between residential buildings and non-residential buildings plus civil engineering infrastructures.

Considering the relative cost of long-distance transport, cement is mostly locally produced and locally consumed^46^. According to the UN comtrade statistics, only 112 Mt cement and clinker was traded internationally in 2018, compared to the total cement production with 4050 Mt. Therefore, in this study we assume all domestic cement demand will be always met by local production.

#### Cement emission projection framework

On the basis of the projected cement demand, the future cement emissions over 2020–2050 can be further estimated by emission factor method. This section describes the approach to project future emissions, which integrates the plant- and country-level calculation methods. See details in Supplementary Methods and Supplementary Figs. 14 and 15.

##### Distribution of new capacity and existing capacity

Considering continued growth in cement demand and the retirement of some existing cement plants, many more cement plants will need to be built. To estimate the distribution of new capacities and existing capacities in response to the growing cement demand, the capacity factor of existing cement plants in one country is assumed to be unchanged. When cement demand in a country increases and exceeds total production capacity of existing cement plants, new capacity is calculated by total cement demand minus cement production from existing plants. Emissions from the new capacity are calculated at the country-level. For the countries witnessing the decreasing cement demand over time where local capacity from existing cement plants is large enough to satisfy the current cement demand, existing cement plants will reduce the capacity equivalently to match the cement demand.

##### Plant-level emissions projections of existing cement plants

When estimate future CO_2_ emissions of existing cement plants, the global plant-level cement database provides essential plant-level information on commission year, capacity and process parameters. The commissioning year determines when the cement plant would be expected to retire (the retirement age is set at 50 years^58,59^). The plant-level kiln type and annual capacity integrated with country-level energy structure and emission factors are the foundation to project future cement plants’ emissions. Also, the plant-level database helps develop mitigation strategies for cement plants. In the Kiln Energy Efficiency Scenario, the plant-level information on commission year, kiln type and capacity is adopted to determine whether to retrofit and the order of retrofitting among the cement plants, making the simulation of retrofitting close to the real life. To be specific, existing cement plants with wet, semi-wet, semi-dry and shaft kilns will be retrofitted to dry kilns linearly before 2030 and the plants with larger cement capacity are assumed to be retrofitted earlier, excluding the old plants that would retire before 2030. The mitigation effects of waste fuels and SCMs on existing cement plants are explored based on the country-level energy structure and clinker-to-cement ratio data.

There are obvious advantages by integrating plant-level database into future cement emissions projection, which helps incorporate greater real-world detail into future emissions projections. To be specific, first, the plant-level information on kiln type gives specific thermal efficiency for each cement plant, which is the foundation of the accurate emissions mitigation assessment. Secondly, the information on plant age and capacity makes it possible to accurately simulate the retirement and newly construction of cement plants and calculate the different emission factors under the four cement production scenarios. As newly built cement plants are more energy-efficient and low-carbon than existing plants, the average emission factor in cement sector of one country will be lower when the share of newly built cement plants grows. Thirdly, the plant-level information on kiln type and capacity makes the simulation of retrofitting close to the real life.

##### Country-level emissions projection of future new cement capacity

Country-specific parameters on cement production, energy intensity, emission factor, clinker-to-cement ratio are adopted to account for the cement emissions. Also, the emission mitigation effects of low carbon measures for newly built cement plants are assessed on country-level.

### Data used in models

#### Historical data to construct global cement database

The basic information is mainly collected from *The 13*^*th*^ *Global Cement Report* by CEMNET and *National Cement Production Line Atlas 2019* published by CCEMENT. Local cement databases for major cement production countries as well as other global databases are also investigated and incorporated in the global cement plants database of this study. See details in Supplementary Methods and Supplementary Fig. 11.

#### Data for the estimation of carbon emissions

The emission factors of calcination, share and carbon content of different fuels are collected from the UNFCCC. The ratio of clinker to cement is from the IEA^11^. The cement production by country is mostly collected from *The 13*^*th*^ *Global Cement Report* by CEMNET, and in part from local databases. See details in Supplementary Table 3.

#### Data for the projection of cement demand

*IIASA* provides the projection for population and GDP data every five years from 2020 to 2050^47,60,61^. Per capita residential floor area is estimated and projected using applied logistic functions relative to GDP per capita^62^ (see details of equation in Supplementary Methods). To give a more accurate projection, the value of some countries is further scaled by per capita residential floor area collected from existing publications. See details of data source in Supplementary Table 7.

#### Data for the estimation of emissions reduction

The technical parameters proposed by IEA^11,59^ are adopted as the input for our scenario analysis, which are more reliable and widely accepted. See details of data source and model assumptions in Supplementary Table 7 and 12.

### Limitations

Due to limited data availability over trade in cement, this study needed to make some simplifying assumptions. For example, we assumed that all domestic cement demand would be met by local production, neglecting the effects of international trade on a country’s cement emissions. The assumption would lead to higher estimates of cement production (as well as CO_2_ emissions from cement) for clinker importing countries such as Ghana, Sri Lanka, and the Phillippines. Future research could address this limitation by taking international trade into account. Integrating economic models (for example, input-output or computable general equilibrium models) would help quantify cement trade volumes, calculate domestic cement product and explore any carbon leakage arising from differential regulation across countries.

Our model designs the future pathways for cement industry by investigating the mitigation effects of four low carbon measures. There is a need to put in more effort into refining the scenario setting, in order to minimize the projection uncertainty. For instance, different types of SCMs are associated with varying emission calculation methods. The use of materials like blast-furnace slag and fly ash doesn’t entail an additional clinkering process^63^, while the adoption of Limestone Calcined Clay Cement (LC3) necessitates an extra calcination step, leading to increased emissions^64^. According to simulation results, the cumulative mitigation effect of LC3 over 2020-2050 is projected to be 14% under the BAU cement production scenario, which is 5% lower than the effect achieved with blast-furnace slag or fly ash. Future work on cement emission modeling could be further improved to be more holistic and inclusive by taking more potential mitigation measures into consideration, such as waste heat recovery^65^, construction and design efficiency improvements^17^, value-chain related mitigation strategies^66^, construction and demolition wastes recycling^67^, etc. As previous researches usually account for the mitigation effect of each measure solely, there lack holistic researches that look into the combined effects from the adoption of different measures and make comparisons between the measures. Emerging radical and disruptive innovative technologies are also suggested to be taken into consideration when designing future cement emission mitigation paths.

The mitigation projection model in this study is established based on the technical feasibility of low carbon measures. The model could be further improved if the economic aspects of the low carbon techniques in cement industry could be considered and integrated. Adoption of the low carbon measures in the construction industry depends upon its relative cost at user level. For each low-carbon measure, the lower economic costs enable the increase in application scale. The integration of economic cost into modeling would help simulate the technology scenarios closer to the real-world and fulfill the understanding of the future technology distribution, thereby having massive consequences on the future low-carbon cement pathways.

Apart from the intensive carbon emitter, the cement has relatively recently been recognized as a potentially important CO_2_ sink^16,28^. The work on cement emission accounting and projection could be further improved and extended through taking into consideration the re-uptake of CO_2_ by cement. The scale of historical CO_2_ absorption occurred along the entire cement cycle has been estimated regionally^68,69^ and globally^70^, with the fraction of re-uptake around 5% of the total CO_2_ emitted during current cement production^70^. The rapid growing cement production in deevloping countries pointed out by this study calls for a proper, holistic projection of the mitigation potential of the sponge effects of the future cement stocks. The database on future country-specific cement demand driven by different infrastructure targets compiled by this study, provide essential information on cement stock for future work.

### Supplementary information

Supplementary Information

### Source data

Source Data

## Data Availability

The numerical results plotted in Figs. 1–3 are provided in the source data with this paper. Our analysis mianly relies on two datasets, each used with permission and/or by license. The first dataset includes plant-level data for global cement industry(excluding China), which we obtained from *The 13th Global Cement Report* and online cement database by CEMNET. The second dataset includes plant-level data for China cement industry, which we obtained from *the National Cement Production Line Atlas 2019* published by CCEMENT. We do not have permission to share the raw data, but we provide sources of the original database. All the data sources and their detailed information are listed in Supplementary Fig. 11, Supplementary Table 3 and Supplementary Table 7. Source data are provided with this paper.

## Code availability

All modeling frameworks in this paper are clarified in the “Methods” section. The relevant codes that were used to project cement demand and emissions are available from the corresponding author on request.
