# Supplementary material for: Projecting future carbon emissions from cement production in developing countries
Source: Nat Commun. 2023 Dec 11;14:8213. doi: 10.1038/s41467-023-43660-x (PMC10713616; doi:10.1038/s41467-023-43660-x)
Supplement: Supplementary file 1 — Supplementary Information [file 41467_2023_43660_MOESM1_ESM.pdf]

Projecting future emissions from cement production in developing countries

Supplementary Items

Supplementary Figures

Here we show eleven supplementary figures to give a more in-depth illustration of the results. Figure S 1 to Supplementary Figure 3 are designed for the first part of the result in the main text- the distribution of global cement emissions. These figures give detailed information on time series cement production and emission intensity for selected countries. The countries are selected as case countries and can represent a wide range of countries in terms of their cement industry. They all have large population size, yet are different in the geological site, economic development and urbanization patterns and have significantly different cement production patterns. Supplementary Figure 4 presents the logistic relationship between GDP per capita and floor area per capita. Figure S 5 and Figure S 6 present the detailed future emission pattern.Supplementary Figure 7 and Supplementary Figure 8 give the evidence for the discussion part of climate target. Supplementary Figure 9 and Supplementary Figure 10 describe the results for the sensitivity analysis. Supplementary Figure 11 to Figure S 16 support the methodology.

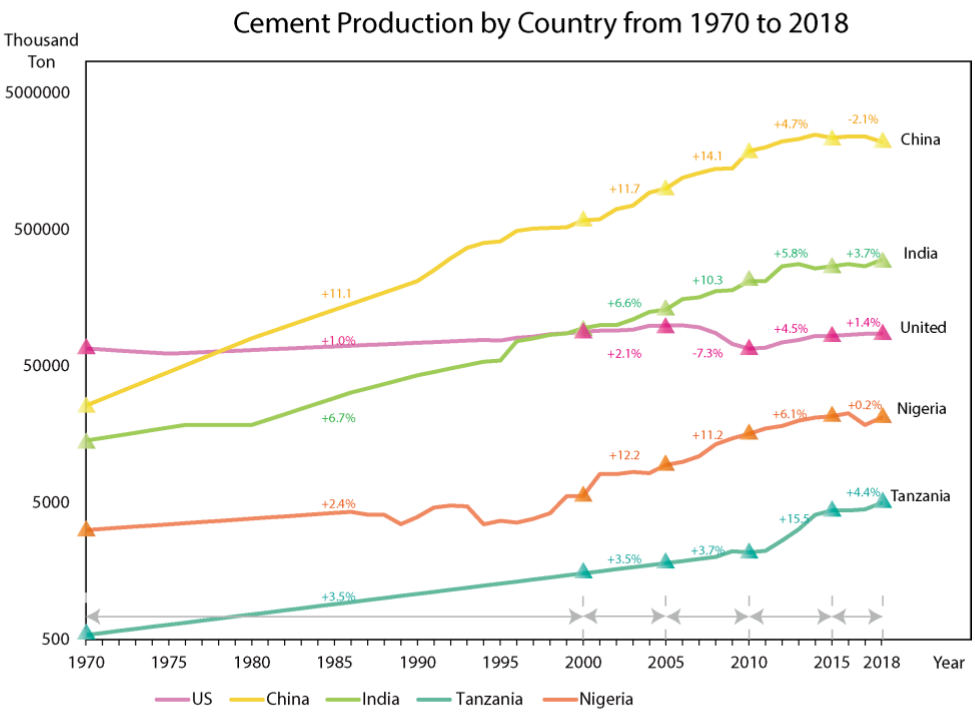

Supplementary Figure 1| Cement production for selected countries from 1970 to 2018. Due to data availability constraints, linear interpolation is adopted to fill the gap in cement production data over 1970-1993. Each number between two triangles indicates the average annual growth rate of cement production during the period. Related to Figure 1.

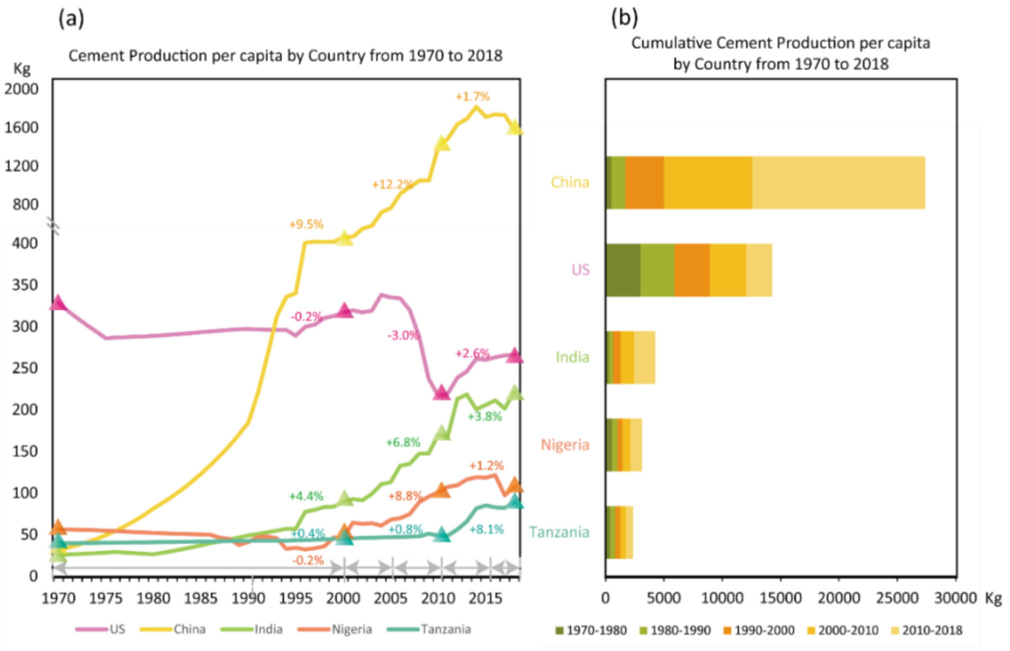

Supplementary Figure 2| Per capita cement production for selected countries from 1970 to 2018. Linear interpolation is used in certain time periods when data is not available. Pane (a) describes the variation of cement production per capita by country over time period, with each number between two triangles indicating the average annual growth rate of cement production per capita during the period. Pane (b) describes the cumulative cement production per capita by country, with the color indicating different decades. Related to Figure 1.

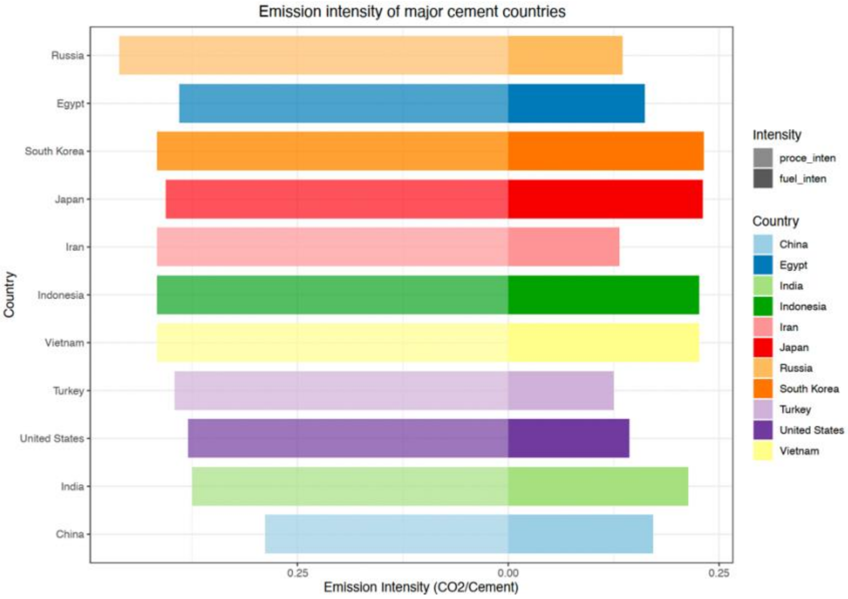

Supplementary Figure 3| Emissions intensity of major cement countries. The light color refers to process-related intensity and the dark color refers to fossil fuel-related intensity. Related to Figure 1.

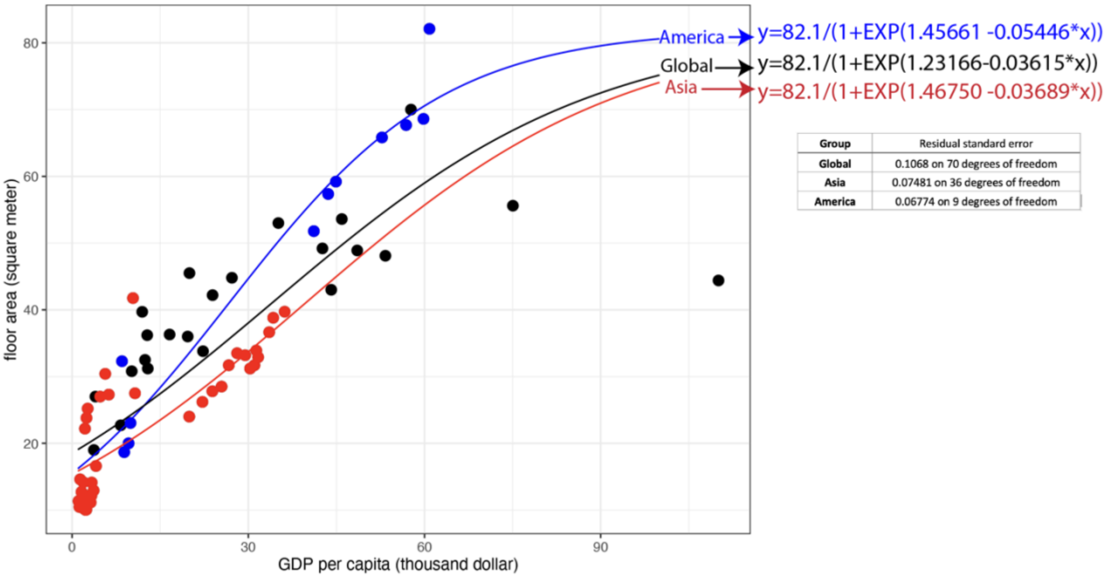

Supplementary Figure 4| The residential floor area per capita as a function of GDP per capita. X-ray correspond to the per capita GDP (2015 constant dollars), and Y-ray correspond to the residential floor area per capita. Each plot refers to one country in one year. Please see the list of countries in Supplementary Table 11. The American countries are in blue and the blue curve refers to the floor area project model for American countries. The Asian countries are in red and the red curve refers to the floor area project model for Asian countries. The black curve refers to the global floor area project model which is fit by all countries worldwide. Due to the data availability, African countries adopt the global model.

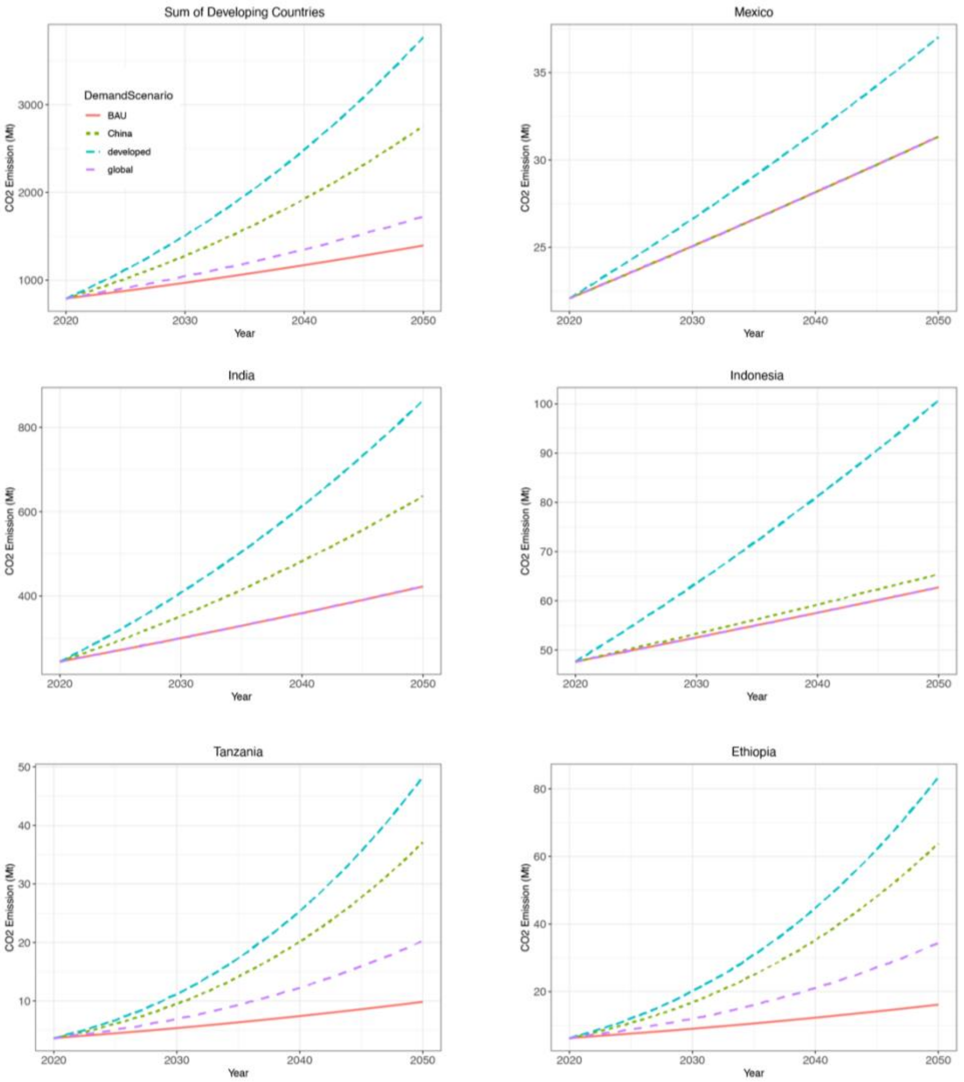

Supplementary Figure 5| Annual emissions of sum of developing countries (except China) and selected countries over 2020-2050. The line corresponds to different cement production scenarios: BAU, global average, China level and developed average scenarios.

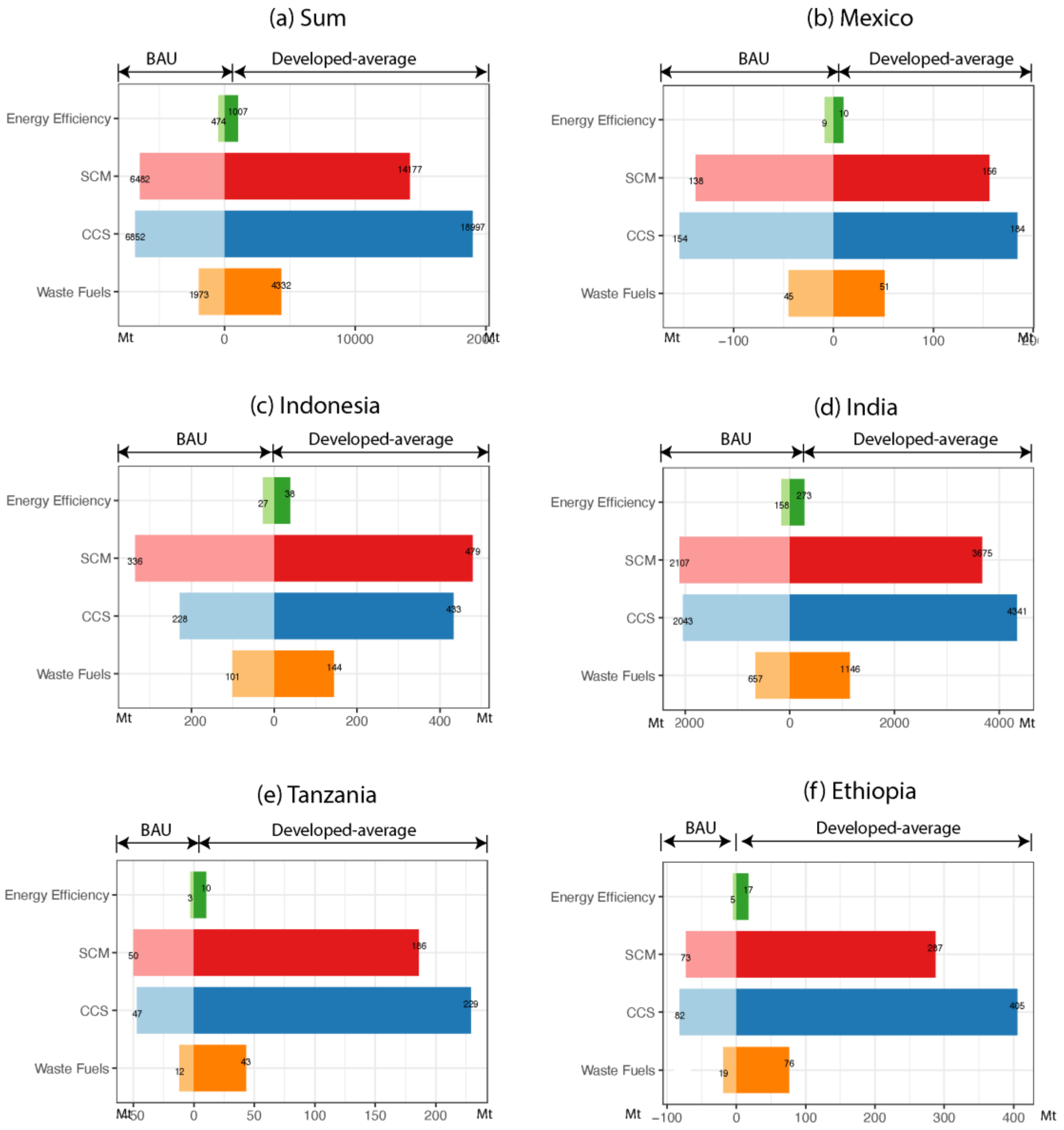

Supplementary Figure 6| The emission reduced by four low-carbon measures under different cement production scenarios. (a), the emissions reduced by four low-carbon measures under BAU and Developed-average cement production scenarios, respectively. (b)-(f), cement emissions of the following selected countries under the same scenario combinations: Mexico (b), Indonesia (c), India (d), Tanzania (e) and Ethiopia (f).

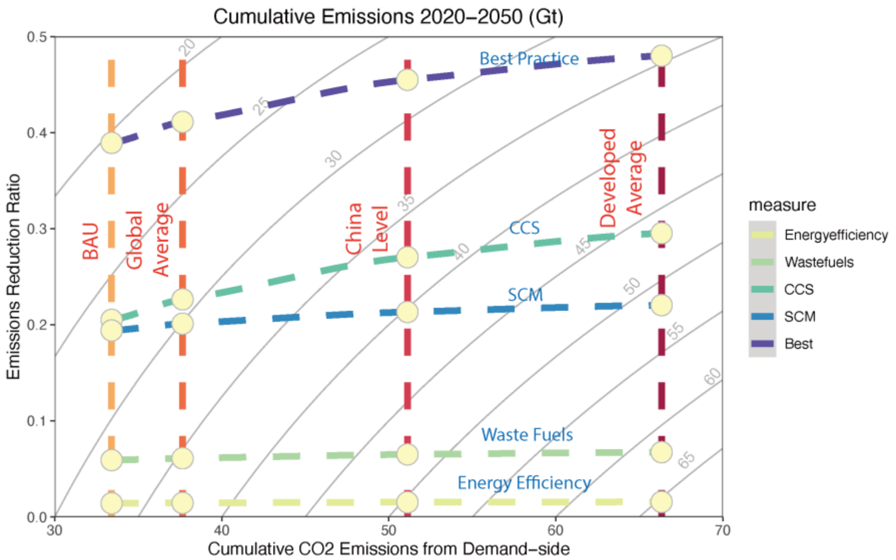

Supplementary Figure 7| Cumulative CO_2 emissions from developing countries (except China) over 2020-2050 (grey lines of the contours) depend on both cement demand and mitigation measures. The dashed vertical lines indicate the cumulative CO_2 emissions driven by the different levels of cement demand needed to expand infrastructure, whereas the dashed horizontal lines indicate the reduction in cumulative emissions resulting from the application of the different mitigation measures. Cumulative CO_2 emissions are therefore calculated by multiplying cumulative emissions from demand by the expression 1- emissions reduction ratio (where the ratio is defined as the absolute reduction in the cumulative cement emissions divided by baseline cumulative cement emissions). The intersection points (the large circles) reflect the combination of cement production levels and emission mitigation scenarios.

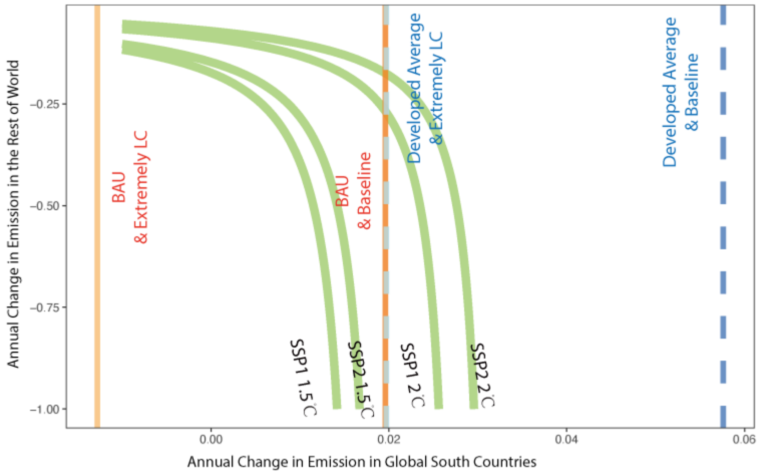

Supplementary Figure 8| Carbon emissions budget for cement. The green lines show the combinations of the annual changes of CO_2 emissions of the two types of countries to reach the 1.5 or 2 ℃ target. The orange solid vertical lines represent annual growth rate of the developing countries (except China) under the BAU cement demand scenarios, with light orange for the extremely LC scenario and dark orange the baseline. The blue dashed vertical lines represent the same for the Developed Average demand scenario.

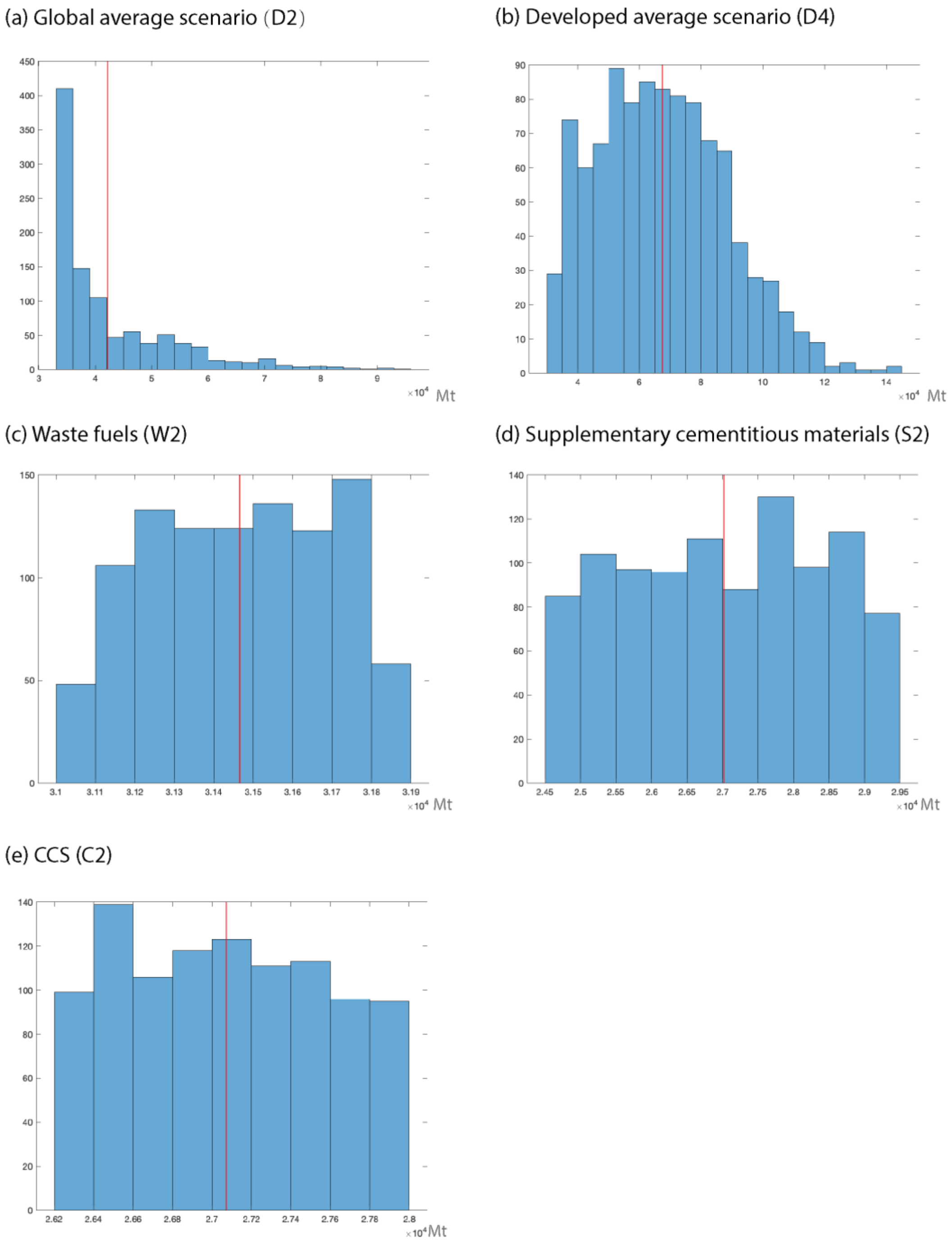

Supplementary Figure 9| The probability distribution bar graph. The x axis refers to the cumulative emissions (Mt) for the sum of developing countries (except China) over 2020-2050. The y axis shows the frequency among 1000 times sampling.

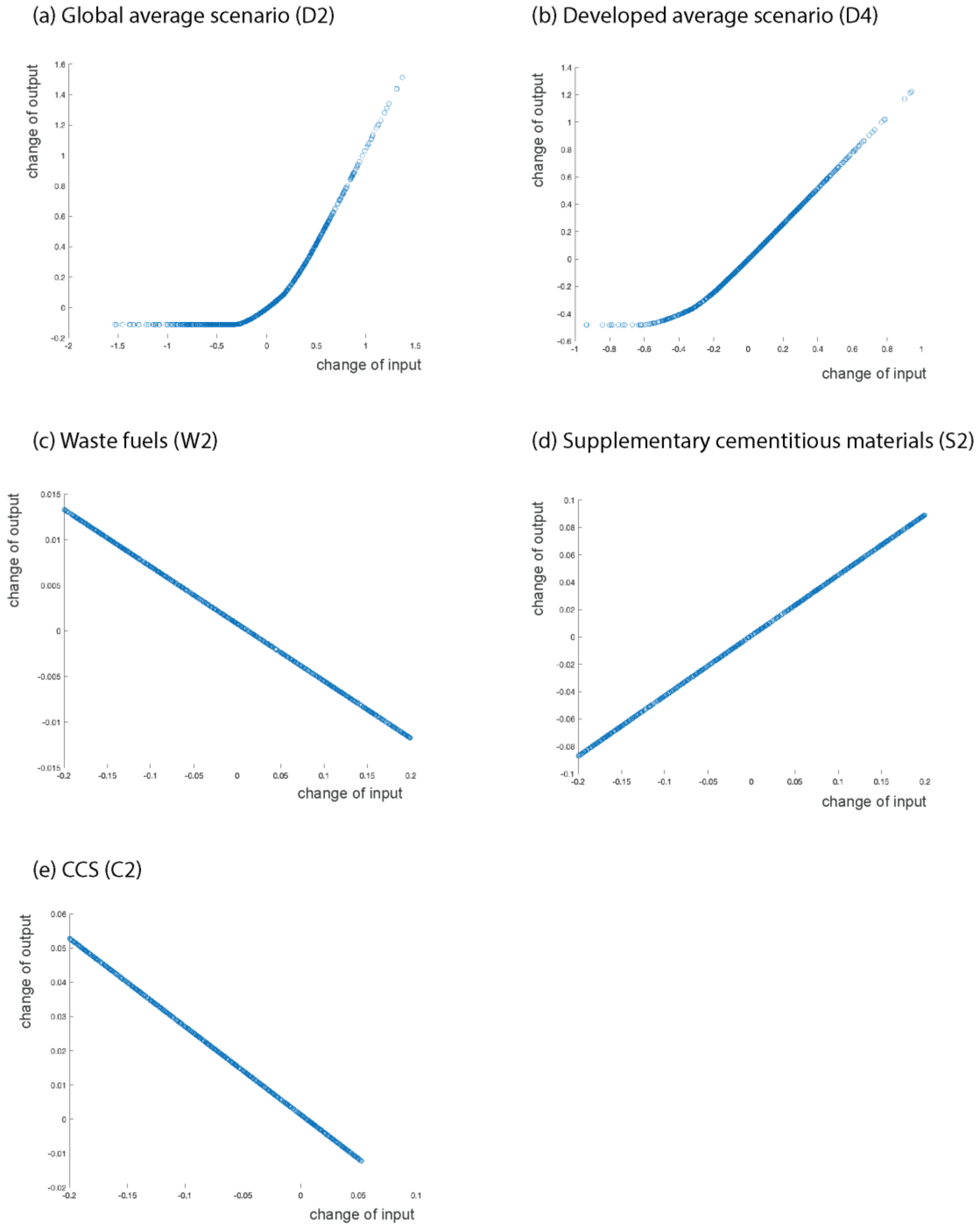

Supplementary Figure 10| Scatter plots for change of input variables (x axis) and change of output (y axis). a-e refer to global average scenario, developed average scenario, waste fuels, supplementary cementitious materials, CCS.

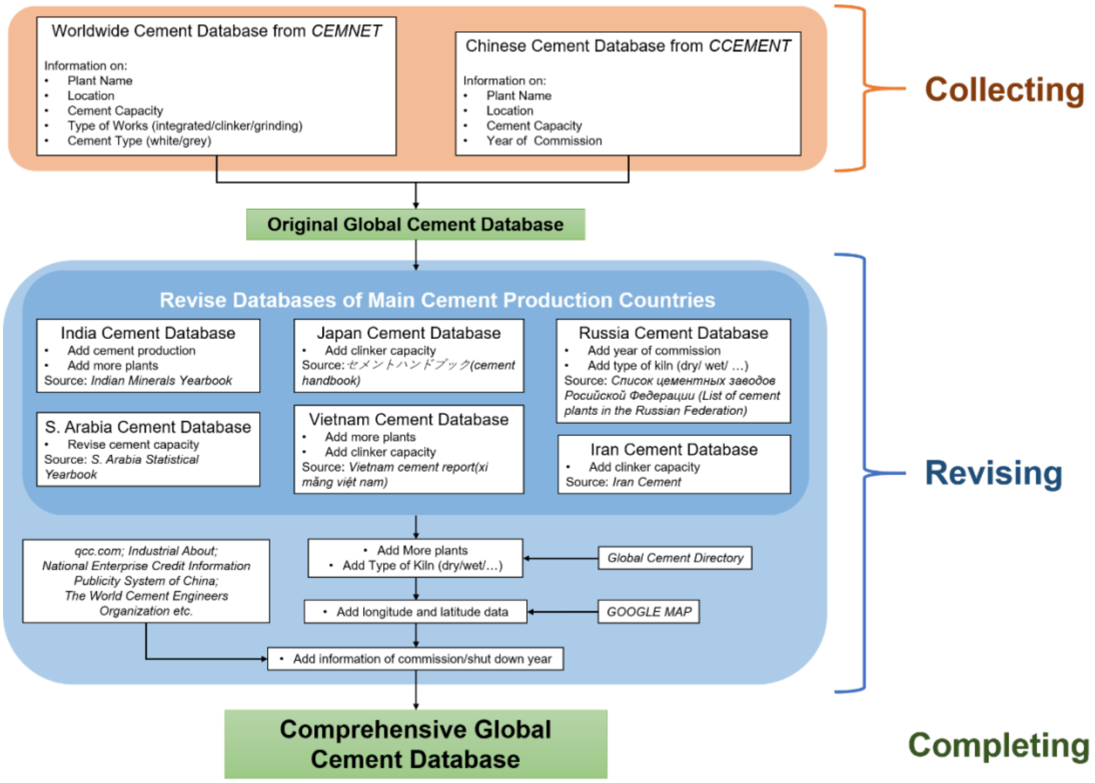

Supplementary Figure 11| The description of construction of global cement database.

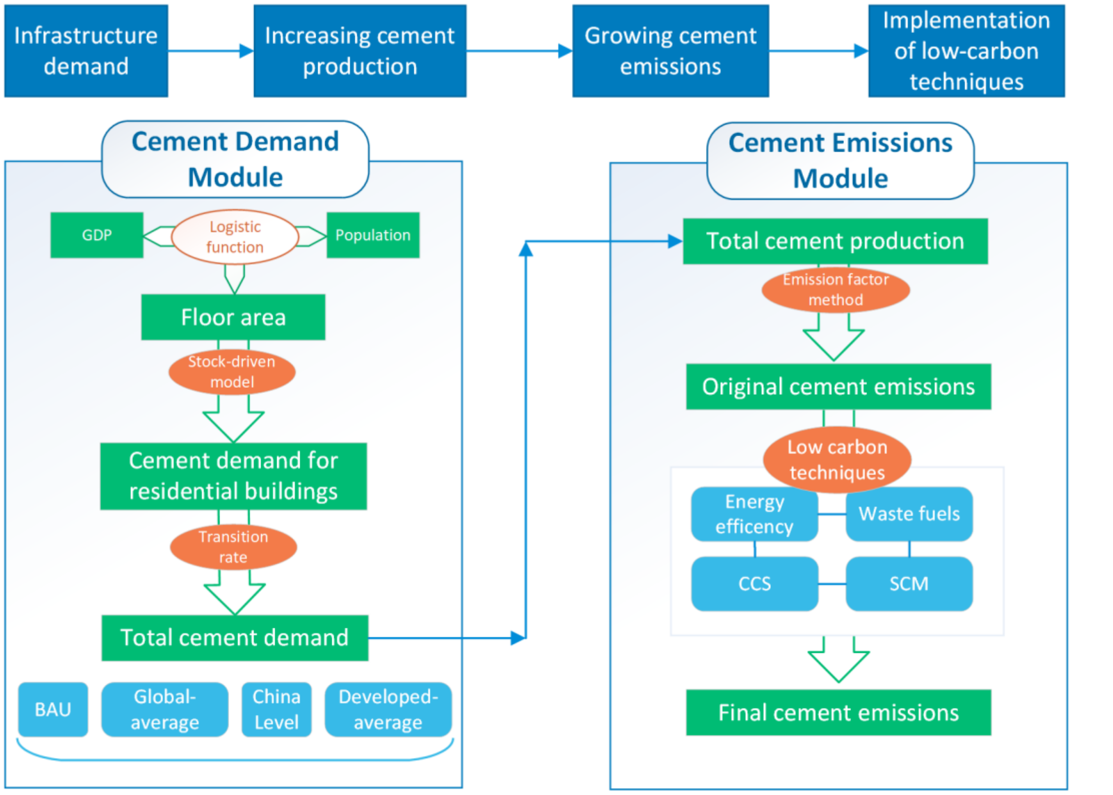

Supplementary Figure 12| The flow diagram for the scenario analysis in future cement emissions.

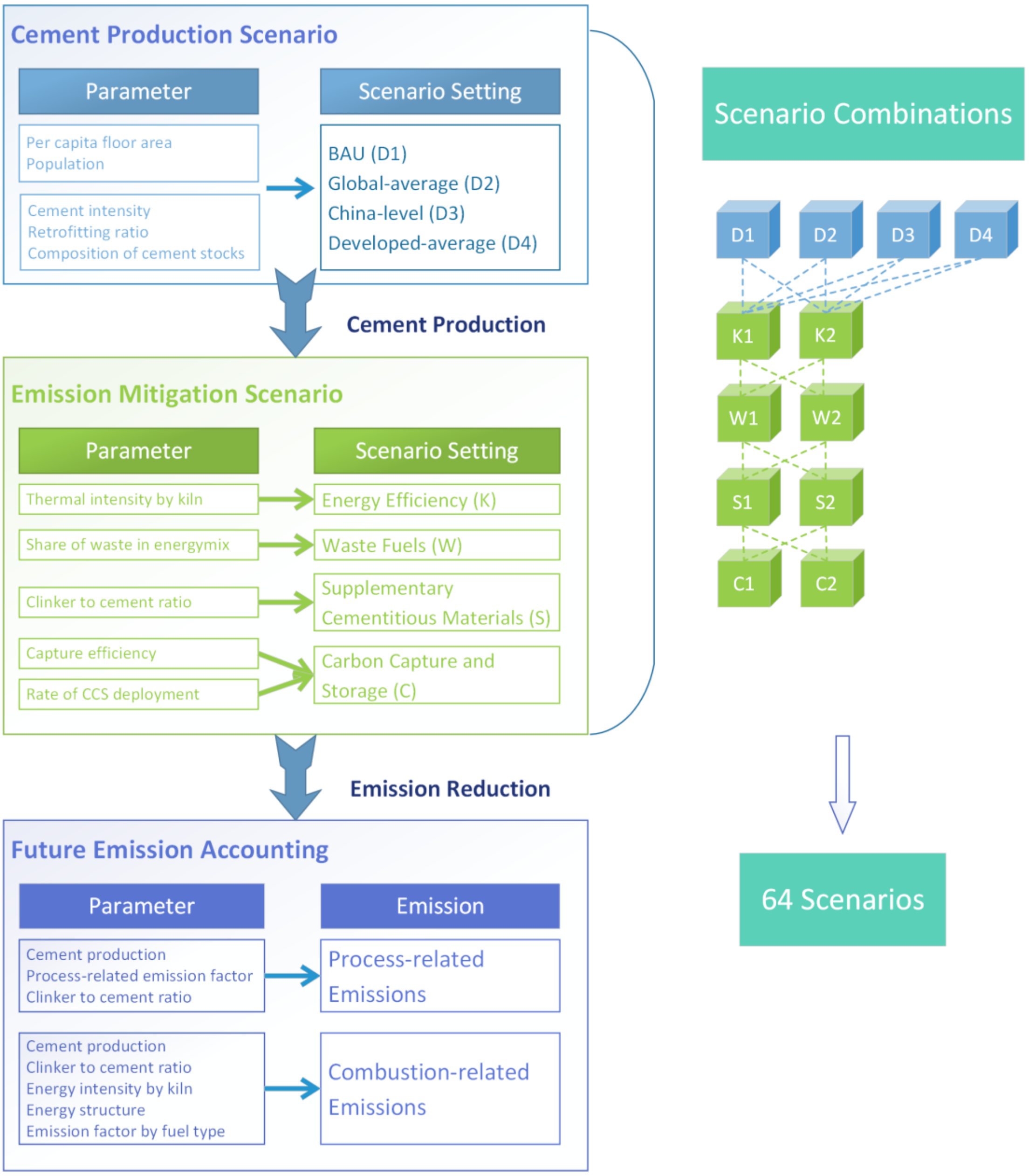

Supplementary Figure 13| The description of variables and outputs in scenario analysis in future cement emissions.

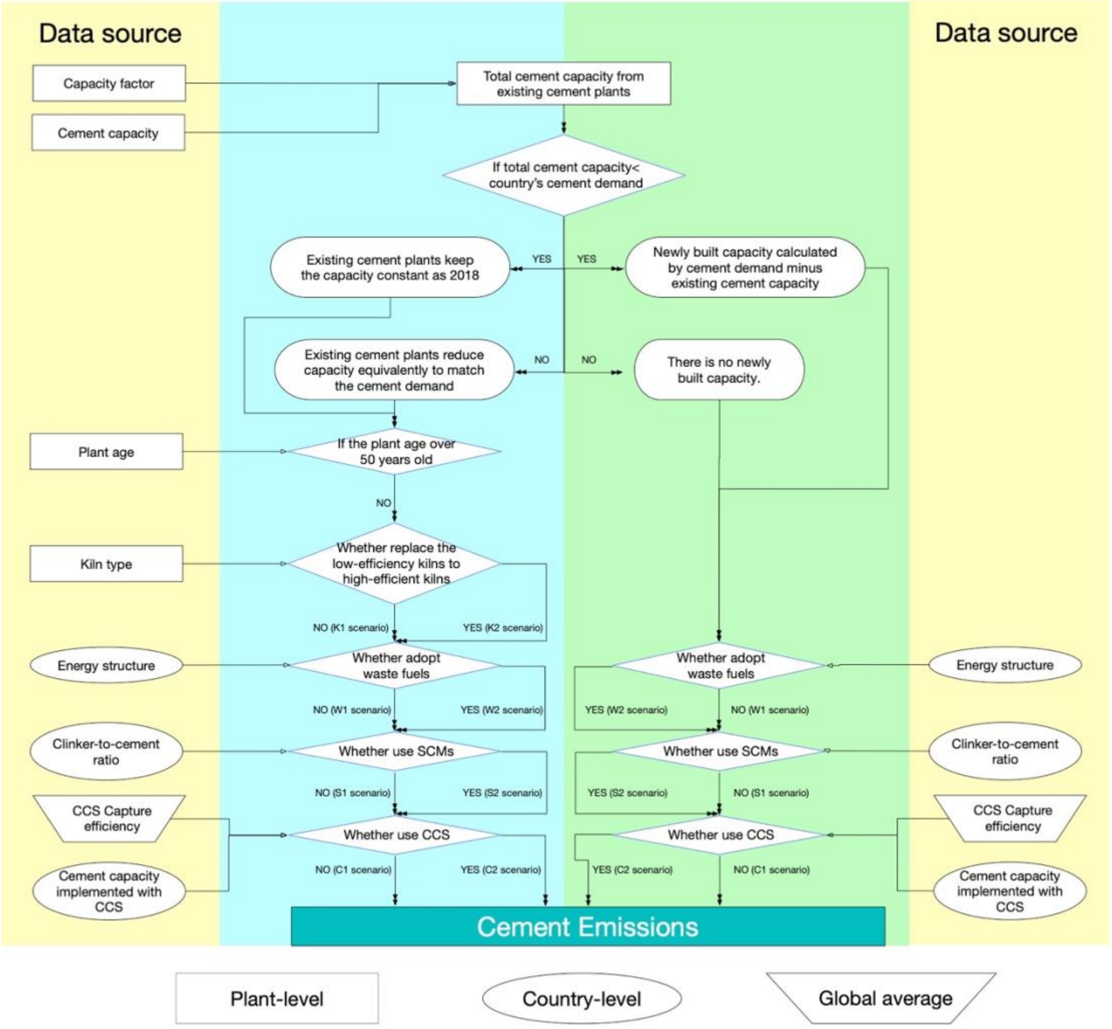

Supplementary Figure 14| The methodological illustration about how we integrate mitigation measures in the emissions accounting framework. The figure describes the key steps in the emission mitigation module and the data sources of the migration strategies.

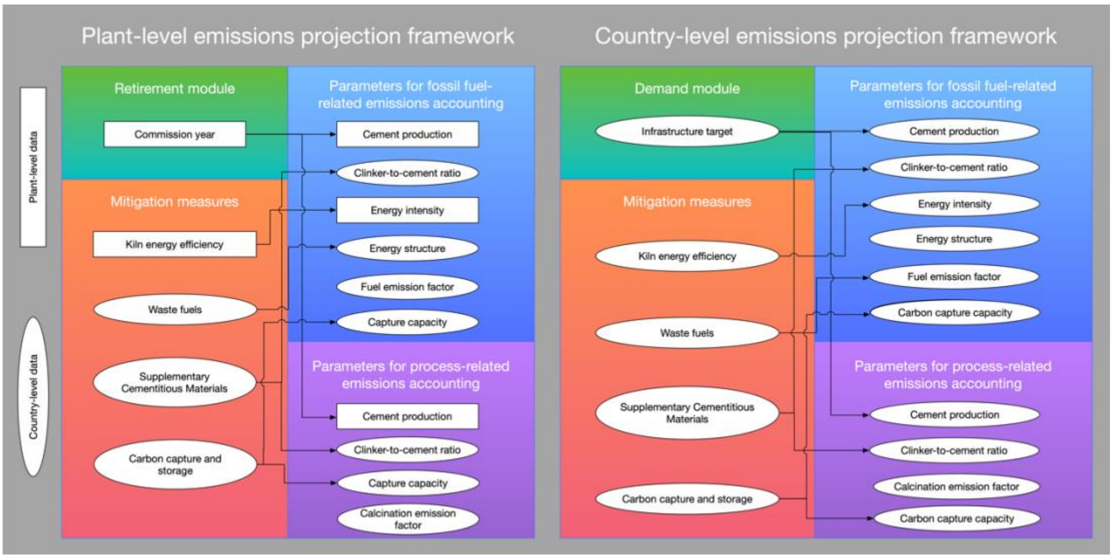

Supplementary Figure 15| The illustration of the integrated plant- and country-level emissions

projection framework. The differences of data availability and accounting approach between plant- and country-level emissions projection framework are showed in the figure. The data availability is presented by the shape of text box. We also show how each measure affects the cement process- and fossil fuel-related emissions by linking the processes (retirement module, demand module and mitigation measures module) to the parameters.

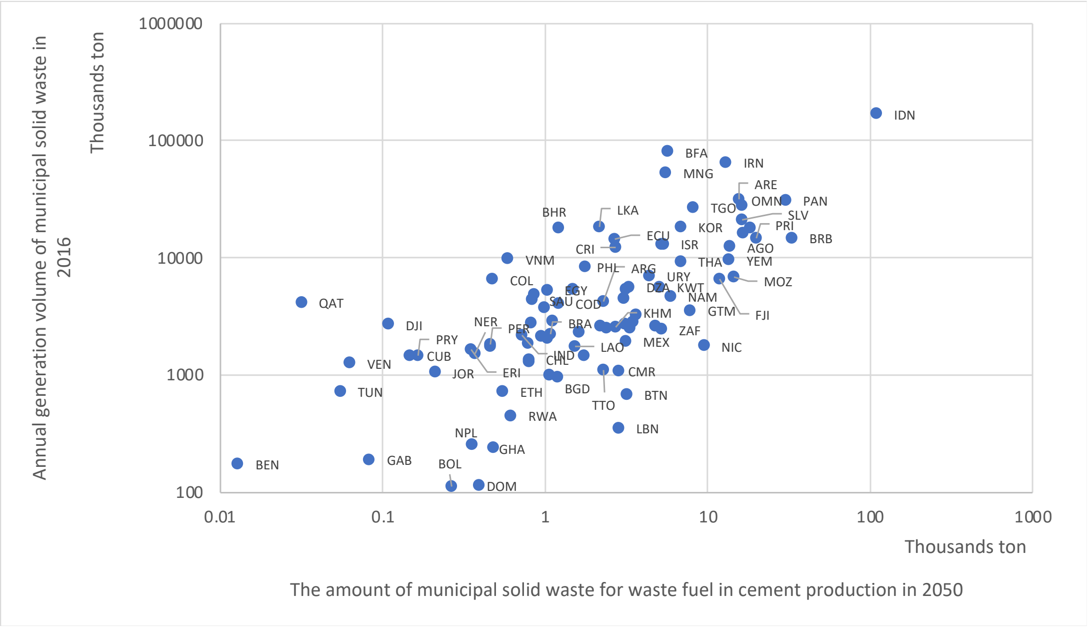

Supplementary Figure 16| The distribution of country-specific waste fuel availability. The waste fuel availability is calculated by dividing the amount of municipal solid waste for waste fuel in cement production in 2050 by annual generation volume of municipal solid waste in 2016. Annual generation volume of municipal solid waste in 2016 is collected from *A global snapshot of solid waste management to 2050*[1]. The municipal solid waste (MSW) used for waste fuel in cement production in 2050 is estimated based on the data of energy intensity, energy structure and waste heat value. The average heating value of MSW is estimated to be around 10 MJ/kg[2,3]. Also, it is assumed that the municipal solid waste will continue rising over 2020-2050[1].

Supplementary Tables

Supplementary Table 1| The compositional structure of global cement capacity in 2018.

Category	Item	Ratio
Region	China	53.5%
	Other Asia	24.5%
	Former Soviet Union	3.8%
	Oceania	0.1%
	Europe	4.9%
	North America	2.4%
	South America	4.7%
	Africa	6.1%

Commission year	Before 1950	6.5%
	1950-1969	8.1%
	1970-1989	9.3%
	1990-1999	6.8%
	2000-2009	34.1%
	2010-2018	30.9%
	Unknown	4.2%
Kiln type	New dry	53.2%
	Dry	37.2%
	Semi-dry	0.4%
	Semi-wet	0.1%
	Wet	2.2%
	Shaft	0.0%
	Unknown	6.9%

Supplementary Table 2| The global cement emissions accounted by literature.

Year	Accounting scale	Results	Sources
2019	process-, energy- and electricity-related	2.3 Gt CO2	Global Efficiency Intelligence [4]
2014	process- and energy-related	2.2 Gt CO2	International Energy Agency[5]
2019	process- and energy-related	2.5 Gt CO2	Chen et al.[6]
2015	process- and energy-related	2.2 ~ 2.9 Gt CO2	Farfan et al.[7]
2018	process- and energy-related	2.1 Gt CO2	This study
2016	process-related	1.3 Gt CO2	Janssens-Maenhout et al.[8]
2016	process-related	1.9 Gt CO2	Le Quéré[9]
2018	process-related	1.50±0.12 Gt CO2	Andrew [10]
2018	process-related	1.4 Gt CO2	This study

Supplementary Table 3| Description of the data source in estimating cement emissions in 2018.

Item	Source
$EF_{calcination,c}$	Value of 0.4964 t CO_2 / t clinker adopted for Chinese plants[11]; other country values based on *National Inventory Submissions* to UNFCCC.
$PRO_{cement,c}$	*China Statistical Bulletin* provides cement production for China in 2018*; USGS* provides cement production of other major countries (US, Brazil, Egypt, India, Indonesia, Iran, Japan, South Korea, Russia, Turkey, Vietnam) in 2018; *Statistics Canada* provides cement production for Canada in 2018; *CEMNET* Database provides cement production in 2018 for 172 other countries.
$R_{clinker\ to\ cement,c}$	Country-specific data published by International Energy Agency[12].
EI_k	Energy intensity for new dry kilns of 3370.19 MJ/t clinker[13]; dry kilns with 3550 MJ/t clinker, wet kilns with 4800 MJ/t clinker, semi-dry kilns with 4350 MJ/t clinker, semi-wet kilns with 4350 MJ/t clinker, draft kilns

	with 4800 MJ/t clinker, based on European Commission [14].
$S_{i,c}$	Country-specific data provided by National Inventory Submissions of UNFCCC.
$EF_{fuel,i,c}$	Emission factors collected from *Emission Factors for Greenhouse Gas Inventories* of US EPA [15].

Supplementary Table 4| Representative countries in each group according to age structure and per capita cement production (ton).

Group	Country	Age structure		Cement production per capita in 2018 **
		The rapid growth period*	The share of cement capacity from rapid growth period	
Group1	United States	Before 1970	67.6%	0.27
	Turkey	Before 1970	36.3%	0.90
	Japan	Before 1970	96.8%	0.44
	South Korea	Before 1970	52.8%	1.07
	Russia	Before 1970	59.6%	0.37
	Brazil	Before 1970	28.8%	0.25
	Germany	Before 1970	77.6%	0.41
Group2	China	(2000, 2010]	64.6%	1.67
	Saudi Arabia	(2000, 2010]	24.2%	1.76
	UAE	(1990,2000]	40.8%	2.24
Group3	India	(2000, 2010]	20.4%	0.20
	Vietnam	(2000, 2010]	38.3%	0.76
	Iran	(2000, 2010]	32.0%	0.70
	Mexico	(1990,2000]	20.8%	0.32
	Pakistan	(1990,2000]	38.2%	0.17
	Thailand	(1990,2000]	39.2%	0.51
	Morocco	(2000, 2010]	26.6%	0.40
Group4	Indonesia	(2010, 2018]	59.4%	0.26
	Egypt	(2010, 2018]	30.9%	0.48
	Nigeria	(2010, 2018]	63.1%	0.11
	Nepal	(2010, 2018]	55.2%	0.31
	Myanmar	(2010, 2018]	67.0%	0.12
	Kenya	(2010, 2018]	59.8%	0.12
	Tanzania	(2010, 2018]	53.2%	0.09

* The rapid growth period refers to the period when majority cement plants were established. The period is divided by every ten years: before 1970, 1971-1980, 1981-1990, 1991-2000, 2001-2010, 2011-2018.

**The country-specific population data is collected from World Bank Database and the country-specific cement production data is collected from global database of CEMNET.

Supplementary Table 5| Listing of the countries that are investigated in scenario analysis.

Country Name	Region	ISO code	Country Name	Region	ISO code
Cameroon	Central Africa	CMR	Mexico	Mexico and Central America	MEX
Chad	Central Africa	TCD	Nicaragua	Mexico and Central America	NIC
Gabon	Central Africa	GAB	Panama	Mexico and Central America	PAN
Republic of Congo	Central Africa	COG	Argentina	South America	ARG
Djibouti	East Africa	DJI	Bolivia	South America	BOL
drc	East Africa	COD	Brazil	South America	BRA
Eritrea	East Africa	ERI	Chile	South America	CHL
Ethiopia	East Africa	ETH	Colombia	South America	COL
Kenya	East Africa	KEN	Ecuador	South America	ECU
Madagascar	East Africa	MDG	Paraguay	South America	PRY
Rwanda	East Africa	RWA	Peru	South America	PER
Tanzania	East Africa	TZA	Uruguay	South America	URY
Uganda	East Africa	UGA	Venezuela	South America	VEN
Algeria	North Africa	DZA	Cambodia	East Asia	KHM
Egypt	North Africa	EGY	Indonesia	East Asia	IDN
Libya	North Africa	LBY	Laos	East Asia	LAO
Morocco	North Africa	MAR	Malaysia	East Asia	MYS
Sudan	North Africa	SDN	Mongolia	East Asia	MNG
Togo	North Africa	TGO	Myanmar	East Asia	MMR
Tunisia	North Africa	TUN	North Korea	East Asia	PRK
Angola	Southern Africa	AGO	Philippines	East Asia	PHL
Malawi	Southern Africa	MWI	South Korea	East Asia	KOR
Mozambique	Southern Africa	MOZ	Thailand	East Asia	THA
Namibia	Southern Africa	NAM	Vietnam	East Asia	VNM
South Africa	Southern Africa	ZAF	Afghanistan	South Asia	AFG
Zambia	Southern Africa	ZMB	Bangladesh	South Asia	BGD
Zimbabwe	Southern Africa	ZWE	Bhutan	South Asia	BTN
Benin	West Africa	BEN	Fiji	South Asia	FJI
Burkina Faso	West Africa	BFA	India	South Asia	IND
Ghana	West Africa	GHA	Iran	South Asia	IRN
Mali	West Africa	MLI	Nepal	South Asia	NPL
Niger	West Africa	NER	Pakistan	South Asia	PAK

Nigeria	West Africa	NGA	Sri Lanka	South Asia	LKA
Senegal	West Africa	SEN	Bahrain	Western Asia	BHR
Syria	Western Asia	SYR	Iraq	Western Asia	IRQ
Barbados	Caribbean	BRB	Israel	Western Asia	ISR
Cuba	Caribbean	CUB	Jordan	Western Asia	JOR
Dominican Republic	Caribbean	DOM	Kuwait	Western Asia	KWT
Haiti	Caribbean	HTI	Lebanon	Western Asia	LBN
Jamaica	Caribbean	JAM	Oman	Western Asia	OMN
Puerto Rico	Caribbean	PRI	Qatar	Western Asia	QAT
Trinidad and Tobago	Caribbean	TTO	Saudi Arabia	Western Asia	SAU
Costa Rica	Mexico and Central America	CRI	Turkey	Western Asia	TUR
El Salvador	Mexico and Central America	SLV	UAE	Western Asia	ARE
Guatemala	Mexico and Central America	GTM	Yemen	Western Asia	YEM
Honduras	Mexico and Central America	HND			

Supplementary Table 6| Comparison of floor area growth rate between IEA and this study.

Country/Region	Annual growth rate of total floor area over 2015-2050 estimated by IEA [16,17]	Annual growth rate of total floor area over 2020-2050 calculated in this study under BAU
India	3.8%	4.0%
Other Asia	2.2%	2.5%
Latin America and Caribbean	2.3%	1.9%
Africa	3.3%	2.8%
Total	2.9%	2.8%

Supplementary Table 7| Descriptions of variables in scenario analysis.

Module	Variable	Source	Level
Cement Demand Module	Per capita floor area	Collected directly as shown in Supplementary Table 11; estimated based on logistic function with per capita GDP when data is not available	Country-specific
	Population	Collected from the IIASA database under SSP2 scenario[18-20]	
	GDP	Collected from the IIASA database under SSP2 scenario[18-20]	

	Cement intensity	Collected from *Global Status Report 2018*[21] (see Supplementary Table 8)	Region-specific
	Composition of cement stocks	Estimated based on the data of major cement producers[22] (see Supplementary Table 9)	
	Demolition rate	Collected from [23-26]	Global average
Cement Emissions Module	Cement production of existing cement plants	In most cases, future cement production of each existing cement plant is calculated by multiplying plant-level cement capacity (from the Global Plant-level Cement Database) by the capacity factor (calculated by Equation (3)). However, for the countries where local production capacity from existing cement plants exceeds the total cement demand of the country, existing cement plants will reduce their capacity equivalently to match the cement demand.	Plant-specific
	Cement production of newly built cement plants	Country's new capacity is calculated by country's cement demand (projected by the Cement Demand Module) minus total cement production of existing plants.	Country-specific
	Clinker to cement ratio	*Cement Technology Roadmap 2018*[12]	Country-specific
	Energy mix	*National Inventory Submissions* of UNFCCC[15]	
	Emission factor of fossil fuels	*Emission Factors for Greenhouse Gas Inventories* of US EPA [15]	
	Thermal intensity	Determined by the kiln type from Global Plant-level Cement Database	Plant-specific
	Cement capacity equipped with CCS	50% of each country's new cement capacity and 10% of each country's installed cement capacity assumed to be implemented with CCS technology	Country-specific
	CCS capture efficiency	Efficiency of carbon capture is set as 95%[27]	Global average
	Retirement year	Determined by the commissioning year found in Global Plant-level Cement Database	Plant-specific

Supplementary Table 8| Data for cement intensity by region.

	Residential cement intensity	Residential cement intensity

	in 2020 (kg/sqm)	in 2050 (kg/sqm)
Central Asia	180	180
Former Soviet Union	180	180
Latin America	180	180
Middle East and North Africa	90	150
North America	60	60
South And Pacific Asia	180	180
Sub-Saharan Africa	90	150
China	180	180
India	180	180

Supplementary Table 9| Transition rate by region.

Region	Transition rate
Asia	2.4
Former Soviet Union	4
Latin America	3
Rest of the world	3

*The transition rate is defined as the newly-built cement stocks of non-residential buildings and civil engineering infrastructures divided by newly-built cement stocks of residential buildings.

Supplementary Table 10| The uncertainty caused by transition rate.

Scenarios	Cumulative cement production over 2020-2050 with constant transition rate (Mt)	Cumulative cement production with transition rate growing by 20% over 2020-2050 (Mt)	Change rate
BAU	55539	60099	8.2%
Global Average scenario	62401	67669	8.4%
China Level scenario	84691	92279	9.0%
Developed Average scenario	106577	116443	9.3%

Supplementary Table 11| The value and data source for per capita floor area of major countries.

Country	Per capita floor area (sqm)	Year	Data source
Austria	48.1	2008	Eurostat
Croatia	36.2	2008	Eurostat
Estonia	36.3	2008	Eurostat
Greece	42.2	2008	Eurostat

Hungary	39.7	2008	Eurostat
Latvia	31.2	2008	Eurostat
Lithuania	32.5	2008	Eurostat
Luxembourg	44.4	2008	Eurostat
Netherlands	53.6	2008	Eurostat
Norway	55.6	2008	Eurostat
Poland	30.8	2008	Eurostat
Portugal	45.5	2008	Eurostat
Romania	22.7	2008	Eurostat
Slovenia	33.8	2008	Eurostat
Sweden	48.9	2008	Eurostat
UK	43	2008	Eurostat
France	53	2009	Eurostat
India	10	2014	[28]
Japan	23	2015	Japan Ministry of Land, Infrastructure, Transport & Tourism
China	40.8	2016	China Premium Database's Household Survey
Vietnam	22.8	2016	Vietnam Ministry of Construction
Australia	70.0	2017	Point2Homes.com & Shrinkthatfootprint.com
Brazil	32.3	2017	Point2Homes.com
Germany	49.2	2017	Eurostat
Malaysia	27.5	2017	Valuation and Property Services Department, UNCHS and the World Bank
Mexico	32.9	2017	Point2Homes.com
Spain	44.8	2017	Eurostat
Sri Lanka	16.6	2017	Valuation and Property Services Department, UNCHS and the World Bank
Thailand	29.7	2017	Valuation and Property Services Department, UNCHS and the World Bank
Czechia	36	2018	Eurostat
Philippines	23	2018	Philippine Statistics Authority
Canada	58.3	2018	Natural Resources Canada
Iran	27	2019	Financial Tribune, Eghtesad Online News
US	82.1	2019	American Housing Survey (AHS)
South Korea	31.7	2018	Statistics Korea
Egypt	19	2017	*Census Data for Egypt*
Tunisia	27	2010	Elaboration d'un Plan pour la Rénovation Thermique et Energétique des Bâtiments Existants en Tunisie
Saudi Arabia	24	2017	Rooftop PV Potential in the Residential Sector of the Kingdom of Saudi Arabia
Cambodia	14.6	2020	Cambodia Socio-Economic Survey

Supplementary Table 12| Summary of the calculation methods and basic assumptions in mitigation scenario setting.

Mitigation measure	Baseline scenario	Mitigation scenario		
	Basic assumptions	Calculation methods	Parameter setting of mitigation measure	Basic assumptions
**Energy efficiency**	• Existing plants will keep constant as usual.<br>• New plants will be equipped with dry kilns.	• Retrofitting to dry kilns with higher energy efficiency will lower the thermal intensity.	• Existing wet, semi-wet, semi-dry and shaft kilns will be retrofitted to dry kilns before 2030.<br>• New cement plants will be built with dry kilns.	• Existing kilns will be retrofitted linearly.<br>• All dry kilns would be implemented with the pre-decomposition kiln.<br>• The cement plants owning large capacity will be retrofitted earlier than those with low capacity.
**Waste fuels**	• The energy structure will keep constant as usual.	• The adoption of waste fuels will directly change the energy structure.	• The share of waste fuels in energy structure will be 30% in 2050[12].	• The ratio of waste fuels would increase linearly from the current level of biomass.<br>• The increasing share of waste fuels in the energy mix leads to the decreasing share of fossil fuels.
**SCMs**	• The clinker to cement ratio will keep constant as usual.	• The adoption of SCMs will directly change the clinker-to-cement ratio.	• The clinker-to-cement ratio will be reduced to 0.60 in 2050[12].	• The clinker-to-cement ratio will linearly decrease from the current level to the target.
**CCS**	• There is no CCS application.	• Calculate the quantity of CO2 captured by multiplying the capture efficiency and the total emissions[7,29]	• The capture efficiency is 95%[30].<br>• 50% of future new cement capacity and 10% of existing cement capacity will be equipped with CCS[31].	• Deployment of CCS starts in 2025[17], and the retrofitting of existing plants would be completed before 2030.<br>• New cement capacity would be equipped with the oxy-fuel technology and existing cement capacity with the post-combustion technology[32].<br>• Cement plants with larger capacity will be retrofitted earlier than those with lower capacity.

Supplementary Table 13| List of assumptions on carbon capture efficiency for CCS technology in cement industry from the literature.

Carbon capture efficiency	CCS Technology	Source
>90%	Amine scrubbing, full oxy-fuel combustion, calcium looping	ref[33]
Assume 40%, 60%, 80% and 100% for different scenarios	Not mentioned	ref[34]
Assume 63%, 85%, 100%	Oxy-fuel combustion, amine scrubbing, calcium looping post-combustion, respectively	ref[35]
Assume 90%	Post-combustion	ref[36]
Assume 90%	Oxy-fuel combustion	ref[37]

Assume 90%	Calcium looping post-combustion	ref[38]
Assume 74% and 61%	Post-combustion and oxy-fuel combustion, respectively	ref[29]
Assume 78% and 61%	Post-combustion and oxy-fuel combustion, respectively	ref[39]
Assume 80%	Not mentioned	ref[40,41]
Assume 86%	Oxy-fuel combustion capture CCS	ref[42]

Supplementary Table 14| List of the uncertain variables included in the simulations and their statistical distributions

Factor	Default	Distribution	Range
**Cement production scenarios**			
**Global-average floor area (sqm/cap)**	27.6	Normal	(μ,σ)=(27.6,13.6)
**Developed-average floor area (sqm/cap)**	47.9	Normal	(μ,σ)=(47.9,14.2)
**Emission mitigation scenarios**			
**Share of waste fuels (%)**	30	Uniform	[24, 36]
**Clinker to cement ratio (%)**	50	Uniform	[40, 60]
**The efficiency of carbon capture (%)**	95	Uniform	[76,100]

Supplementary Table 15| Descriptive statistics of output cumulative emissions (Mt) over 2020-2050 under different variables.

Factor	Mean	Median	Percentile (10%, 90%)	Range (min, max)
**Cement production scenarios**				
**Global-average**	42147	37777	(33429, 57691)	(33429,94594)
**Developed-average**	67450	65901	(39840, 95280)	(33429,143070)
**Emission mitigation scenarios**				
**Waste fuels**	31466	31469	(31155, 31772)	(31065, 31852)
**Supplementary Cementitious Materials (SCM)**	27018	27029	(25063, 28880)	(24581, 29324)
**Carbon capture and storage (CCS)**	27071	27061	(26402,27794)	(26230, 27956)

Supplementary Table 16| The uncertainty of adopting region-specific floor area projection model.

Model	Ratio of change
Global-average model	[-17%, +18%]
America-specific model	[-27%, +23%]
Asia-specific model	[-19%, +21%]

Supplementary Table 17| The change of thermal energy intensity by kiln type over 1990-2016.

Kiln type	1990-2000	2000-2010	2010-2016
Dry kiln with preheater and precalciner kiln	-5.9%	-0.4%	0.0%
Dry kiln with preheater without precalciner	-4.5%	0.3%	1.0%
Semi-wet/semi-dry kiln	-5.6%	1.2%	7.3%
Wet/Shaft kiln	-4.9%	-0.4%	-1.3%

Source: World Business Council for Sustainable Development (WBCSD) [43]

Supplementary Notes

Note S 1| Descriptions of countries shown in the Figure 2 in the main text.
We find that these countries can represent a wide range of developing countries in terms of future cement emissions pattern. These countries have large population, but different performances in economic development, geological sites, and cement industry.

Mexico Mexico is located in southern America. It is the world's 13th-largest country by area and the 10th-most-populous country. As a newly industrialized and developing country, high in the Human Development Index, its large economy and population, cultural influence, and steady democratization make Mexico a regional and middle power which is also identified as an emerging power.
Mexico is the 11th largest cement producing country in the world producing 51 million tonne (Mt) of cement in 2021. Around 87 % of the energy used in Mexico's cement industry is fossil fuels dominated by petroleum coke, natural gas and coal.
India India is in South Asia, connected by land to countries such as Pakistan, China, Nepal, and Bhutan, etc. According to the latest census by the Bureau of Statistics of India, the country has a population of 1.38 billion, making it the second most populous country in the world after China. India is the sixth largest economy in the world in terms of nominal GDP, with a GDP of $2.623 billion in current prices in 2020[44]. Due to its huge population, its per capita GDP is only US$1,900, which is at the level of a low-income country in the world.
India is the second largest producer of cement in the world. It accounts for more than 7% of the global installed capacity. India has a lot of potential for development in the infrastructure and construction sector and the cement sector is expected to largely benefit from it. Some of the recent initiatives, such as development of 98 smart cities, is expected to provide a major boost to the sector.
Aided by suitable Government foreign policies, several foreign players such as Lafarge-Holcim, Heidelberg Cement, and Vicat have invested in the country in the recent past. A significant factor which aids the growth of this sector is the ready availability of raw materials for making cement, such as limestone and coal.
Indonesia The Republic of Indonesia, is located in southeastern Asia, straddling the equator, and bordering Papua New Guinea, East Timor, and Malaysia. Indonesia is the world's largest archipelago country, consisting of approximately 17,508 islands between the Pacific and Indian

Oceans, with a land area of approximately 190.4 km^2. In 2021, Indonesia has a total population of 268 million, ranking fourth in the world. Indonesia has enjoyed relatively steady economic growth since the 1960s, making significant progress in agriculture, energy extraction and textiles, making it the largest economy in the Association of Southeast Asian Nations (ASEAN). In 2020, Indonesia's gross domestic product calculated at comparable prices is US$1.06 trillion, ranking 15th in the world. Although its total GDP is large, Indonesia's per capita GDP is still below the global average, making it a low-and middle-income country in the world.

Cement consumption is still low in Indonesia with per capita cement production at approximately 300 kilograms. This figure is much lower than cement consumption in its peers Vietnam. A low per capita cement consumption figure implies that infrastructure development is still underdeveloped in Southeast Asia's largest economy. The cement sector's long-term picture is positive with the continuation of a rapidly expanding middle class. With rising per capita GDP people want to live in a better house. The country's total installed production capacity expanded from 37.8 million tons in 2010 to over 100 million tons in 2016, while domestic sales surged from 40 million tons to an estimated 60 million tons over the same period. The Indonesian government, under the leadership of President Joko Widodo, has given more attention to infrastructure development in order to boost the country's economic growth in a productive way. Funds allocated to infrastructure spending has risen markedly in recent years.

Tanzania Tanzania is an East African country located in the Great Lakes Region of Africa. Tanzania's economy has grown at an average annual rate of 6.3% from 2010 to 2018. In 2019, Tanzania's GDP was US$ 63.2 billion, and its population was 58 million.

Since 2014, Tanzania has dramatically increased cement production. Between 2018 and 2019 alone, cement production increased from 4.5 million metric tons to roughly 6.5 million metric tons an increase of 44.5 percent.

Ethiopia Ethiopia is a landlocked country located in the Horn of Africa. It is the 13th-most populous country in the world, the 2nd-most populous in Africa after Nigeria, and the most populated landlocked country on Earth.

Ethiopia's cement industry has witnessed substantial growth in the past decade With nearly 16.5 million tonnes of cement capacity and 10% average growth in annual consumption, Ethiopia is among the top cement producers in sub-Saharan Africa. Only Nigeria and South Africa rival it.

Supplementary Methods

Construction of Global Cement Database

Historical Data to Construct Global Cement Database

Global cement database is constructed mainly based on the global database of CEMNET. *The 13th Global Cement Report* and online database of cement plants published by CEMNET contain listings of 2189 cement plants worldwide (excluding China), and give latest cement plant information including plant name, geographical location, cement capacity, type of works and cement type. For China, the *National Cement Production Line Atlas 2019* published by *CCEMENT* provides information on Chinese cement plants, including plant name, geographical location, capacity and year of commissioning.

In order to provide more specific and accurate data, other global and local databases have been consulted in compiling the comprehensive global cement database. Local databases for

leading global cement producing countries including India, Russia, Saudi Arabia, Vietnam, Iran and Japan are collected and supplemented in the original global cement database (see details in Supplementary Figure 11). Data from the *Global Cement Directory 2019* published by *Global Cement* is adopted to provide more detailed cement plant information and supplement the information on kiln types.

The final comprehensive global cement plant database contains 3094 cement plants, of which there are 3020 integrated plants and 74 clinker plants. As this study considers only direct (Scope 1) emissions from cement plants including process- and energy-related emissions. The database gives information on plant names, location sites, operators, host countries, cement capacities, type of works (integrated or clinker) for all cement plants, and year of commissioning, cement type (grey or white) and type of kiln (dry, semi-dry, semi-wet, wet, shaft and new dry kiln) for the majority of plants, and clinker capacity and cement production for some plants.

Estimation of CO_2 emissions from cement

Process-related Carbon Emissions

Process-related carbon dioxide emissions represent the CO_2 emitted during the calcination of raw meal, in which the limestone is heated to produce lime and carbon dioxide. Existing studies tend to use clinker production to calculate the cement process-related emissions, to achieve more accurate emission accounts for the cement industry[45]. Therefore, in this study, the process-related carbon emissions are estimated as clinker production multiplied by the corresponding emission factors; see Equation (1)[46]:

$$E_{process,a,t} = AD_{clk,a,t} * EF_{calcination,c} \quad (1)$$

where $AD_{clk,a,y}$ refers to the clinker production of the plant *a* in year *t*; $EF_{calcination,c}$ represents the country-level emission factor for the clinker production during the calcination of raw meal, that is, the CO_2 emitted during per unit production of clinker.

In the absence of clinker production data, we estimated this by using clinker to cement ratios and capacity factors ($CF_{c,t}$, that is, the utilization rates) on country-specific, shown as Equation (2).

$$AD_{clk,a,t} = CAP_{cmt,a,t} * CF_{c,t} * R_{clk\ to\ cmt,c} \quad (2)$$

In the above equation, $CAP_{cmt,a,t}$ refer to cement capacity of the plant *a* and $R_{clk\ to\ cmt,c}$ represents the clinker-to-cement ratio of the country or region *c*. The $CF_{c,t}$ is calculated as following:

$$CF_{c,t} = \frac{PRO_{cmt,c,t}}{CAP_{cmt,c,t}} \quad (3)$$

Where $CAP_{cmt,c,t}$ is the total cement production capacity in a country or region *c* in year *t*; $PRO_{cmt,c,t}$ represents the cement production in country or region *c*. If $PRO_{cmt,c}$ is absent, the global average capacity factor is adopted.

Energy-related Emissions

The direct energy-related CO_2 emissions are estimated using Equation(4): in year *t*

$$E_{combustion,a,t} = AD_{clk,a} * EI_k * \sum(S_{i,c} * EF_{fuel,i,c}) \quad (4)$$

Where EI_k denotes energy intensity (J/kg clinker) of kiln type, *k*; $S_{i,c}$, and $EF_{fuel,i,c}$ represent the share, and emission factor of the ith type of fuel in country or region *c* where the plant *a* is located; and i represents different types of fossil fuel used to supply energy, including oil, coal

and natural gas.

Categorization of countries based on the plant-level cement database

This study categorized the global countries into four groups according to their age structure of cement plant and per capita cement production. We divide the period by every ten years and identify the rapid growing period of each country when the majority of cement capacity were built (as shown in Table S 4). The countries that witness the rapid growing of cement capacity before 1990 are categorized into the Group 1, while the countries whose rapid growing period is 2011-2018 are categorized into the Group 4. The rest countries are categorized as Group 2 or Group 3, which are differentiated by the per capita cement production. To be specific, China, Saudi Arabia and the United Arab Emirates are defined as Group 2 countries, because their cement production per capita (1.67, 1.76, 2.24 ton, respectively) is significantly higher than other countries with the average of 0.49 ton.

Scenario analysis on Future Cement Emissions

Description of Scenario Analysis

To evaluate future cement CO_2 emissions in developing countries, we propose two scenario sets (tier 1 and tier 2) that correspond to the different level of cement production and emissions mitigation options respectively. More specifically, Tier 1 scenarios represent the estimated cement production based on different levels of cement production to expand the built environment. Tier 2 scenarios present the commonly discussed low carbon measures in cement industry, which consist of thermal efficiency improvement, waste fuels, carbon capture and storage and supplementary cementitious materials[47]. We treat each scenario set as an individual variable in the model, such that we will have five variables. We quantify the mitigation potentials by using different combinations of five variables and to yield 64 scenarios (*D, K, W, S, C, see details in the following*).

We focus on all developing countries (except for China), which are listed in *World Economic Situation Prospects* published by United Nations [48]. China is excluded because its cement production already peaked in 2014 [45,49] and more attention should be paid to countries where the vast majority of future growth will occur and which have been relatively neglected.

Tier 1: Cement Production Scenarios.

In this study, projection of cement demand is linked to per capita floor area [50] and population growth. Considering the relative cost of long-distance transport, cement is mostly locally produced and locally consumed [51]. Therefore, in this study we assume all domestic cement demand will be always met by local production. When total cement demand in the country or region exceeds total production capacity of existing cement plants, more cement plants are built to satisfy the increasing cement demand. We define four scenarios of cement production labeled D1 through D4, where D stands for ‘Potential Cement Demand’, representing varying degrees of possible future infrastructure growth. The moderate scenario D1 corresponds to economic development under the SSP2[19], while the most ambitious D4 is close to the housing condition under the SSP5[19]. The average per capita floor area for all developing countries under

D4 (48 sqm) is close to the average level estimated by SSP5 (46 sqm)[18-20]. To be specific,

BAU(D1): anticipates that infrastructure will grow according to the speed of GDP per capita by country under the SSP2. In general, the average growth rate of per capita floor area across developing countries is 62% over 2020-2050.

Global Average (D2): building on the BAU D1, all countries which do not currently meet a level of 29 m^2 floor area per person will accelerate linearly construction to achieve this target in 2050. This scenario means that, some countries will accelerate the expansion of construction in order to catch up with those in the same group that are more advanced so that in 2050 all countries will reach the average level of global countries' housing conditions in 2020. The average growth rate of per capita floor area for all developing countries is 86% over 2020-2050.

China Level (D3): similarly, extending D2, all countries that do not currently meet the level of 40 m^2 floor area per person will accelerate to achieve this target in 2050. In this scenario means that all countries will reach the 2020 level of China in housing conditions. The average growth rate of per capita floor area for all developing countries is 121% over 2020-2050.

Developed Average (D4): finally, extending ambition even further, countries that do not currently meet the level of 47 m^2 floor area per person will accelerate to achieve this target by 2050. In this scenario, all countries will reach the average 2020 level of developed countries in housing conditions. The average growth rate of per capita floor area for all developing countries is 151% over 2020-2050.

Tier 2: Mitigation Scenarios

To date, proposed roadmaps for carbon reduction in the cement sector [7,47,52-54] present different decarbonization pathways. The most prominent approaches include: energy efficiency improvement, fuel substitution, replacing the clinker with cementitious materials, increasing production of blended cements, and removing CO_2 from the flue gas. Existing literature provides important evidence-based parameters. We decide to adopt the technical parameters proposed by IEA [12,31] as the input for our scenario analysis, which are more reliable and widely accepted. Also, the mitigations options described by Miller et al. are also fully considered when setting the parameters[47]. Energy penalties of low carbon measures are not considered in emission mitigation assessment due to the emission accounting scope.

We consider four types of low-carbon solutions in order to analyze their emissions mitigation potential, including carbon capture and storage (CCS) technologies, supplementary cementitious materials (SCMs), using waste fuels and improving kiln energy efficiency.

We treat each scenario set as an individual variable in the model, such that we will have four variables in these scenario sets (*K, W, S, C*). The baseline emission refers to the mitigation scenario of the combination of measures $K_1W_1S_1C_1$ and the extremely low carbon refers to the mitigation scenario $K_2W_2S_2C_2$.

Scenario Set 1: Kiln Energy Efficiency (K).

Energy-efficient technologies can be divided into two types according to the stage of the process. One type is used during raw materials preparation and the finishing of cement products, including measures such as substitution of ball mills, efficient transportation systems

and energy-efficient separators. The other type is used during clinker production, including refractory improvements in the kiln, energy management and process control systems, improvements in the kiln combustion system, clinker cooler, etc. The literature designs the scenario of energy efficient technology with retrofitting kilns by different time, for instance, phasing out all shaft kilns 2020 for the reference scenario, 2015 for the efficiency scenario, and 2011 for the best practice scenario[55].

In our study, this scenario set corresponds to improving kilns' energy efficiency to improve energy performance levels when economically available.

K1: existing cement plants will keep constant as usual and all newly-built cement plants will be equipped with dry kilns. K1 is in line with the current situation.

K2: existing kilns that are not planned to retire before 2050 will be retrofitted to dry kilns before 2030 linearly. Newly-built cement plants will be equipped with dry kilns. All dry kilns would be implemented with the pre-decomposition kiln to increase the efficiency.

Scenario Set 2: Waste Fuels (W)

Fuel-switching processes aim to replace carbon-based thermal sources (such as coal) with greener (lower carbon) thermal sources such as natural gas, biomass or biogenic fuels. The fuel used in a kiln contributes approximately 40% of CO_2 emissions. Ideally, if an almost-zero carbon emitting fuel were used in place of a carbon-based fuel, the emissions could be reduced by almost 40%. The cement industry could use a variety of delivered waste materials to provide heat for its kilns, including old tires (whole or processed), municipal solid waste, scrap fabrics, paints, and inks. Most such fuels are principally derived from the onsite production processes and cost little or nothing to use, lowering overall fuel costs by displacing purchased fuels. Using solid waste fuels also decreases the volume of waste disposal needed, lowering disposal cost for manufacturers. According to the reference carbon emissions reduction scenario proposed by IEA, the share of alternative fuels will grow to 30% by 2050, which include biomass, biogenic and non-biogenic waste sources. Since this study only accounts for the fossil fuel-related emissions in terms of CO_2 emissions generated from combustion, biomass and waste are considered neutral.

However, alternative fuels depend on availability of feedstock, climate and location. Alternative fuels could also affect product quality and refractory lining in cement kiln as they can retain impurities or pollutants if not treated and prepared carefully[56]. Also, it should be noticed that adoption of waste fuels might require additional thermal energy input. A lower calorific value as well as high chlorine content will possibly increase the specific fuel energy demand per tonne of clinker, thus comes with energy penalties[32]. In this study, future alternative fuels is assumed to be selected based on the adequate calorific values as well as other different criteria such as physical criteria (e.g. potential for air entrainment), chemical criteria (e.g. chlorine, sulphur, alkali and phosphate content), to ensure that kiln operation and product quality meet business-as-usual standards[57].

W1: without adoption of alternative fuels.

W2: use 30% alternative fuels until 2050[12]. The ratio of alternative fuels in the energy mix is assumed to increase linearly from the current level. Alternative fuels include biomass and waste[5]. However, due to the missing country-specific waste ratio data, we simplify the method by adopting biomass ratio as the alternative fuel ratio at the current level. The data

on country-specific biomass ratio is collected from National Inventory Submissions of UNFCCC. The increasing share of alternative fuels in the energy mix is achieved by reducing the share of fossil fuels. The energy structure of fossil fuels is assumed to remain constant in each country over 2020-2050.

Scenario Set 3: Supplementary Cementitious Materials (S).

Fly ash, blast furnace slag and silica fume are three well-known examples of cement replacement materials that are in use today. The decreasing clinker-to-cement ratio will be needed to get on track with the low-carbon cement roadmap. There exists great uncertainty in the proportion of cement replacement that would be possible. One estimate is that 25-35% of Ordinary Portland Cement can be substituted with fly ash[56]. Habert et al. assumed a clinker share of 50% as a technical minimum limit[58]. Similarly, UNEP proposed that up to 50% clinker displacement is possible through optimized combinations of calcined clay and ground limestone as cement constituents without affecting cement properties[59]. To be specific, Limestone Calcined Clay Cement (LC3)-type substitution with clinker factors as low as 50% reach similar mechanical performances as using ordinary Portland cement[60,61]. It is a promising type of cement that is similar to currently commercial cements and so might face lower barriers to commercialization than other novel cement formulations[62].

This study mainly considers the cement properties and mechanical performances when choosing the feasible share of SCMs in cement. The materials are not considered to be a limiting factor due to the high availability for materials used in calcined clay and to extensive amounts of industrial waste or byproducts that could be viable as the solid precursor to geopolymers [63,64].

Regarding the energy penalty of SCMs, the use of SCMs such as blast-furnace slag, fly ash, etc., does not involve an additional clinkering process[65], and the additional electrical energy demand required to grind the SCMs[32] is out of the emission accounting scope of this study. Whereas, the adoption of LC3 requires additional calcination process for the clay, which generates additional emissions and increases energy consumption. The energy intensity of calcined clay is 2.7 GJ/t[66]. According to simulation results, under the BAU cement production scenario, the cumulative mitigation effect of LC3 over 2020-2050 would be 14% under BAU cement production scenario, compared to the effect with 19% achieved by blast-furnace slag or fly ash. In the main text, we did not specify the types of SCMs, and overlooked the emissions induced by the calcination process for the clay.

S1: assume no change in the country-specific clinker to cement ratio;

S2: assume clinker to cement ratio will be 0.50 in 2050 and assume linearly decreasing from the existing clinker to cement ratio for each country from 2021.

Scenario Set 4: Carbon capture and storage (CCS) (C).

CCS is a combination of technologies designed to prevent the release of CO_2 generated through conventional power generation and industrial production processes by injecting the CO_2 in suitable underground storage reservoirs. As for the deployment of CCS, IEA assumes that, globally, 50% of future new capacity will be large kilns (i.e., >2Mt per annum), with CCS equipment [31]. Furthermore, existing cement plants are also expected to be retrofitted with CCS[5,67,68], despite the high economic costs and technical challenges of retrofitting [27]. According

to the European Cement Research Academy and Cement Sustainability Initiative[32], 10% of existing kiln capacity could be equipped with post-combustion technologies although kilns with a capacity of less than 2500 tonnes per day would not be equipped with CO_2 capture technologies due to high costs. In line with these studies, we assume that 50% of future new cement capacity and 10% of existing cement capacity would be implemented with CCS technology. Larger capacity cement plants will be retrofitted earlier than those with smaller capacity.

There are two main types of CO_2 capture technologies that can be applied in the cement industry: post combustion and oxy-fuel techniques. Post-combustion carbon capture involves the separation of CO_2 from cement kiln flue gas and stands out as a potentially promising carbon capture technology for existing cement plants from the perspective of cost[32,69]. As end-of-pipe technologies do not require significant integration with the core process other than re-routing of the flue gas, it could be expected that the retrofit period is aligned as much as possible with a routinely scheduled production stop for maintenance to minimize the economic impact of retrofitting[27]. By contrast, oxygen-based combustion in cement kilns will lead to reduced nitrogen content that does not have to be heated up, which improves fuel efficiency and provides a relatively low-cost option for CO_2 abatement in cement plants compared to other technologies[12,31,70,71]. Thus, we assume that new cement capacity would be equipped with oxy-fuel technology and existing cement kilns would be retrofitted to be equipped with post-combustion technologies.

As for capture efficiency, the assumptions used in each of the analyzed cases is different, and so are the results. For instance, Farfan et al. assumed that, the efficiencies of carbon capture are set at 60% in process-related emissions before 2030, and 70% and 80% for 2040 and 2050, respectively[7]; Zhou et al. assumed a fixed proportion of 85% capture rate for direct emissions for all scenarios[72]; Miller et al. assumed capture rate with 90% for amine scrubbing and calcium looping techniques[47]; Hills et al. expected that the capture efficiency would be >= 90% for amine scrubbing, calcium looping and oxy-fuel techniques[33]; IEA proposed that oxy-fuel techniques could account for greater shares of cumulative carbon captured CO_2 emissions by 2050 globally in contrast with post-combustion, based on current knowledge of the techno-economic performance[12,31], whose capture yields can theoretically reach 90-99%[12]. This study adopted the capture efficiency with 95% which is acknowledged by the experts in the CCS field [27,33,34].

CCS technology incurs an energy penalty. For example, considerable heat is required to regenerate the absorbent if Mono Ethanol Amine (MEA) is used for post-combustion capture CO_2 from flue gas. However, the heat required for the CCS technology can to be provided by electric heaters and/or by using waste heat recovery system[73-75], which would be excluded from the scope of emissions accounting in this study. According to previous studies[74], the CO_2 avoided ratio (the net reduction of CO2 emissions per unit of net output, compared to a reference plant without CO2 capture [76]) for both oxy-fuel and MEA post-combustion capture technology are the same as their CO_2 capture rate (CO_2 captured divided by CO_2 generated with capture). Therefore, the energy penalty of CCS is negligible when only considering direct process- and energy-related emissions.

C1: no application of CCS.

C2: The global deployment of CO_2 capture for permanent storage in the cement sector are planned to start in 2025[17]. The efficiency of carbon capture is set as 95%. 50% of future

new cement capacity and 10% of existing cement capacity will be implemented with CCS technology.

Combination of each parameter of the 5 variables above means we will have 4 * 2 * 2 * 2 * 2 = 64 scenarios.

Projection of future cement demand

This section illustrates the framework to estimate the country-specific cement demand from 2020 to 2050.

Estimation of floor area. The per capita floor area measures the basic human need for shelter and will be a principal factor of rising materials demand for buildings[77,78]. Therefore, we use the per capita floor area as the proxy to estimate the cement demand in this study. The country-specific floor area of residential buildings is estimated using applied logistic functions relative to GDP per capita[79].

First, the actual data for per capita floor area of major countries is collected from local sources as well as open-access databases, as shown in Supplementary Table 11. We try to include the latest floor area data for as many countries as possible. Time series for per capita GDP is collected from the World Bank database.

Second, the relationship between per capita floor area and per capita GDP in the corresponding year (2015 constant Dollars) is modeled using a logistic function[79] (as shown in Supplementary Figure 4). We assume that there exists a positive correlation between the two indices and that the growth of buildings would slow as its stock nears the saturation levels reached in developed countries. Considering the region's similarity in population, urbanization, economic level, etc., existing studies project the future energy and material consumption for building based on region-level assumptions and models[5,80]. Therefore, we establish the region-specific GDP-floor area function (see), which increases the accuracy compared to the global average model adopted in the previous studies[7]. To be specific, we simulate the GDP-floor area function for American and Asian countries respectively. As for African countries, the global average model is adopted due to the local very poor data. According to the simulation results, the floor area of America is usually higher than the global average. It is understandable that America has lower population and larger per capita land area than other regions. Whereas, the per capita floor area of Asia is generally lower than the global average at various economic levels, which could be explained by the high population density in Asia.

Third, per capita floor area ($ar_{c,t}$) in 2019 for countries is estimated by using applied logistic functions relative to GDP per capita ($GDPpc_{c,t}$) in 2019. The region-specific floor area project functions are shown below.

$$ar_{c,t,x} = \begin{cases} 82.1 \div \left\{1 + e^{\left(-0.03615 \times GDPpc_{c,t} + 1.23166\right)}\right\}, x \in \ global\ countries \\ 82.1 \div \left\{1 + e^{\left(-0.05446 \times GDPpc_{c,t} + 1.45661\right)}\right\}, x \in \ American\ countries \\ 82.1 \div \left\{1 + e^{\left(-0.03689 \times GDPpc_{c,t} + 1.46750\right)}\right\}, x \in \ Asian\ countries \end{cases} \quad (5)$$

In this step, we also calculate the average levels of global countries and developed countries

in per capita floor area in 2019, which are the input of model in cement demand scenario analysis.

However, there still exists some uncertainties. The actual data for per capita floor area that we collected is mostly for high-income countries already at high levels. The lack of wide range of countries makes the regression less reliable for the countries with low level of GDP per capita.

The projection of future floor area. Country-specific per capita floor area in 2050 under the BAU scenario (D1) is estimated based on the projected GDP per capita in 2050, by using the logistic functions above. The data needed to estimate country-specific GDP per capita in 2050 is collected from the IIASA database under SSP2[18-20]. We divided the total growth into annual growth averagely, assuming linear growth of floor area per capita over time.

Higher housing demand in D2 to D4 corresponds to greater cement production. To fill the gap, the annual increment of per capita floor area (ar), which is set as constant in the BAU scenario, is assumed to grow linearly between 2020-2050 in the global average scenario. The calculation for China level and developed average scenarios is similar to that of global average scenario.

The mathematical equations used to estimate the total floor area of residential buildings in country/region *c* in year *t* is described below.

$$SR_{c,t} = P_{c,t} * ar_{c,t} \quad (6)$$

where $SR_{c,t}$ is the total floor area of the residential building stock, $P_{c,t}$ is the population of area and $ar_{c,t}$ is the per capita floor area of residential building. The projections for country-specific population every five years over 2020-2050 is taken from the IIASA database under SSP2[18-20]. Linear interpolation is used for those time periods where data are not available.

Estimation of cement demand. The analytical framework to project cement demand includes two sub systems: buildings and civil engineering. The demand for buildings can be divided into two parts- residential and non-residential buildings. Firstly, we adopt the model developed by Hong et al. [81] to estimate cement demand for residential buildings. This model taking floor area as the proxy is essentially grounded on a stock-driven model. The stock-driven model was introduced as an alternative method for simultaneously forecasting resource demand by Müller[82] in 2006, which has now been widely used in the material flow analysis community to discuss with social metabolism and climate change[83-86].

$$N_{c,t,t-1} = SR_{c,t} - SR_{c,t-1} + D_{c,t,t-1} \quad (7)$$

where $N_{i,t,t-n}$ is the floor area of newly built residential building in region *c* in year *t*, and $D_{c,t,t-n}$ is the demolished residential building because buildings will, of course, be dismantled after their service lifetime.

We estimate $D_{i,t}$ by demolition rate (dr_i), which can be expressed as following. Due to problems with data availability, in this research, we adopt the demolition rate with 0.5% for all countries, which is calculated for China and is widely acknowledged and applied[23-25]. Except for India, we adopt the demolition rate with 1.43%, which is estimated by the ratio of buildings over 80 years old[23,26].

$$D_{c,t} = SR_c * dr_c \quad (8)$$

Limited data availability for demolition rates at the national level may limit the accuracy of our cement demand projection model. We conduct a sensitivity analysis of cement demand to the demolition rate. Previous studies find that in high building turnover scenarios, the demolition

ratio may increase by a factor of 1.5 above historical rates [87]. However, even if we assume that demolition rates are increased by a factor of 1.5, total cement demand over 2020-2050 under the BAU scenario from developing countries would only rise by 5%. Therefore, despite the data limitations, based on these sensitivity results we believe that the uncertainty in the demolition rate does not significantly affect the overall findings of our study.

Total cement demand for residential buildings in country or region *c* in year *t* can be expressed as following, multiplying the cement intensity of residential buildings $CIR_{i,t}$ and newly built residential building floor area.

$$CR_{c,t} = \left[SR_{c,t} - SR_{c,t-1*}(1 - dr_c)\right] * CIR_{c,t} \quad (9)$$

In addition to the residential buildings, non-residential buildings include all buildings not intended for private occupancy whether on a permanent basis or not; for example, buildings used for institutional, commercial or industrial purposes. It is also underlined to include infrastructure in future assessment[88]. However, it is difficult to account for non-residential buildings as well as infrastructure stock directly. The detailed data that can be used to directly estimate cement demands, such as per capita floor area for non-residential buildings and cement intensity for transport infrastructure, were not available. Therefore, the indirect way built up a relationship between cement demand from residential building and those from other sectors by assuming varying ratios for them in different countries based on existing studies[78], so that cement demands from those other sectors are calculated. For instance, Yang estimated that per capita construction area of non-residential buildings is 80% of that in residential buildings in European countries[89]; Shi et al. adopted this value with 80% for China[77]; Cao et al. adopted the split ratios of the building sector in China during 1970-2013 are around 75% and ratios of infrastructure sector around 20%[78]. Therefore, this research simplifies the accounting method by assuming a region-specific ratio between residential and non-residential buildings and civil engineering. Existing literature provides the mix of in-use cement stocks between residential buildings, non-residential buildings and civil engineering for the 15 largest cement producers[22]. According to this literature, we assume the transition rate of residential building and the others for Asia with 2.4, Former Soviet Union with 4, Latin America with 3 and rest of the world with 3. To be specific, For Latin America, we have data for Mexico and Brazil and they could be used as a first estimate; for Asia, we have Turkey, Iran, India and China and use the average level of them as the estimate; we get the data for Former Soviet Union directly; as for Africa, we adopt the split ratios for the rest of world.

$$CO_{c,t} = CR_{c,t} * tr_{c,t} \quad (10)$$

where $CO_{c,t}$ is the cement demand of the non-residential building and civil engineering in country/region c in year t, $tr_{c,t}$ is the transition rate.

Then the total cement demand of country/region c in year t ($C_{c,t}$) can be expressed as following:

$$C_{c,t,origin} = CO_{c,t} + CR_{c,t} \quad (11)$$

Despite the good agreement in region-specific cement demand between our study and IEA[12], there still exists uncertainty between the theoretical and actual value of cement demand of each country because most parameters used in the method is region-specific due to the data availability. Thus, this study further adopts a country-specific correction ratio (cr_c) to scale the value of total cement demand in country or region *c* in year *t*.

$$cr_c = C_{c,2020,actual} \div C_{c,2020,estimated} \quad (12)$$

$$C_{c,t,correct} = C_{c,t,origin} * cr_c \quad (13)$$

Estimation of future cement CO_2 emissions under low carbon measures

This section describes the framework to project future cement emissions, which integrates the plant- and country-level calculation methods.

Plant-level emissions projections. To estimate future CO_2 emissions of existing cement plants, the global plant-level cement database is adopted to provides basic plant-level information on commission year, capacity and process parameters. Our approach to projecting future plant-level emissions can be divided into the following steps.
First, the commissioning year is used to determine when the cement plant would be expected to retire. We set the retirement age at 50 years, which is relatively long[90]. For example, IEA previously assumed the lifetime of cement kilns was in the range of 30-50 years[31].
Second, the cement production of each plant is determined by the installed capacity and capacity factor in most cases. We assume that the capacity factors ($\mathrm{CF_{c,t}}$) of cement plants remain constant over time when the demand of cement demand in the region exhibits an upward trend. By contrast, cement plants will reduce the capacity factors uniformly in response to a declining regional demand for cement.
Third, operating cement plants will adopt low carbon techniques according to the scenario setting, which largely reduce cement emissions. To be specific we explore three approaches to existing cement plants: (i) retrofitting low-efficiency kilns contribute to emissions mitigation by reducing the energy intensity of cement plants ($\mathrm{EI_k}$); (ii) using waste fuels as an input will change the composition of energy sources ($\mathrm{S_{i,c}}$) and drive this shift towards less carbon-intensive energy sources and (iii) incorporating SCMs will help decrease cement emissions by reducing clinker-to-cement ratio ($\mathrm{R_{clk\ to\ cmt,c}}$). As for the kiln upgrading, the kiln type of each plant is used to decide whether to retrofit and the commission year is used to determine the plausibility and timing of retrofitting the facilities. To be specific, under the energy efficiency scenario, semi-wet, semi-dry, wet, draft kilns will be retrofitted to dry kilns considering their lower energy efficiency, and all kilns would be implemented with the pre-decomposition kiln to increase the efficiency. The cement plants owning large capacity will be retrofitted earlier than those with low capacity, considering the economic cost. The plants that are assumed to retire before 2050 will not be considered appropriate for kiln upgrading. The effects of adopting waste fuels and SCMs are simulated based on the country-level data of energy structure and clinker-to-cement ratio. Regarding the waste fuels, it is assumed that local municipal solid waste will be sufficient in each country in the near future. We demonstrate the availability of waste fuels by comparing the recent country-specific annual generation volume of municipal solid waste and the future amount of municipal solid waste needed for fuel in cement production (shown in Figure S 16). Regarding the SCMs, there is strong evidence that the materials are not a limiting factor due to the high availability for materials used in calcined clay and to extensive amounts of industrial waste or byproducts that could be viable as the solid precursor to geopolymers[63,64].

Country-level emissions projection. Considering continued growth in cement demand and

the retirement of some existing cement plants, many more cement plants will need to be built and emissions from these newly installed facilities are calculated at the country level. Country-specific parameters on cement production, energy intensity, emission factor, clinker-to-cement ratio are adopted to account for the cement emissions. Furthermore, we explore four approaches to newly built cement plants: (i) adopting high-efficiency kilns; (ii) using waste fuels; (iii) incorporating SCMs; and (iv) incorporating CCS. The emission mitigation effects of four approaches are assessed on country-level. When assessing the effects of high-efficiency kilns, it is assumed that all newly built cement plants would be retrofitted with new dry kilns with the energy intensity of 3370.19 MJ/t clinker[13]. The approach of the waste fuel and SCMs mitigation effects assessment is same to the plant-level projection. For the CCS deployment, we adopt the country-specific CCS installed capacity and global average CCS capture efficiency to estimate the mitigation effects. The CCS installed capacity is calculated by multiplying newly built cement capacity and CCS implementation rate. This study assumes that oxy-fuel technology would be adopted as it improves fuel efficiency and provides a relatively low-cost option for CO2 abatement in cement plants compared to other technologies[12,31,70,71]. Oxy-fuel technologies not only capture fuel-derived CO2 emissions, but also the large proportion emitted by the raw meal calcination. Then, the quantity of CO2 captured is calculated by multiplying (1-capture efficiency factor) and the total emission amount, which is also applied by other researches[7,29].

Sensitivity Analysis

We conduct sensitivity analysis on key scenario assumptions.

The first step is assuming probability distributions foreach input variable. Previous studies usually obtain the distribution from the literature or by assuming a uniform distribution with a variation range of, say, ±20%, 30% or 100% [91-93]. In this study, we assume a uniform distribution with a ±20% uncertainty range for the scenarios of low-carbon measures, while the uncertainty range of variables in cement production scenario is set assuming the normal distribution of per capita floor area with the real data. The distributional characteristics of these variables are listed in Supplementary Table 14.In terms of the cement production scenario set, we choose the global average and developed average scenarios to investigate the sensitivity of future cement production. The China level scenario is excluded in sensitivity analysis, because the uncertainty in this scenario mainly comes from the statistical bias of activity data which is quite low. In the emission mitigation scenario sets, the potential variation in kiln efficiency improvement is not studied, because it is markedly smaller than three other low carbon technologies. We performed Monte Carlo Analysis with 1000 iterations by varying each variable (random sampling) while holding other variables constant.

The uncertainty of the outputs (cumulative emissions over 2020-2050) was represented in Supplementary Table 15. To evaluate the variables' sensitivity, scatter plots and probability distribution figure are also used as below (see Supplementary Figure 9 and Supplementary Figure 10). Scatter plot is a useful tool for rapid determination of the relation between inputs and outputs[94]. If any variable has a considerable effect on any dependent variable, then a discernible pattern would appear in the corresponding scatter plot.

Linear relationship between inputs and output was observed in all these plots except the global average scenario. In the global average scenario, cumulative emissions do not decrease linearly with the decrease of average floor area. This is because with global-average floor area decreasing, more countries have an average floor area larger than global-average floor area. Their future cement demand is therefore assumed to be constant, making cumulative emissions not sensitive to the decrease of global-average floor area. This phenomenon also explains the reason why the cumulative emissions at the 10% are the same as min value of the range. Among three low carbon measures, the output is most sensitive to the supplementary cementitious materials. More specifically, cumulative emissions change -8% when the clinker to cement ratio in cement decreased by 20%. In contrast, the sensitivity level of waste fuels is the lowest. The cumulative emissions changes -1% when the share of waste fuels in energy mix grows by 20%. The supplementary cementitious materials have different variation trends in comparison to CCS and waste fuels, because the higher clinker to cement ratio corresponds to the lower appliance of SCMs.

Supplementary References

1. Kaza, S., Yao, L., Bhada-Tata, P. & Van Woerden, F. *What a waste 2.0: a global snapshot of solid waste management to 2050*. (World Bank Publications, 2018).
2. Malinauskaite, J. *et al.* Municipal solid waste management and waste-to-energy in the context of a circular economy and energy recycling in Europe. *Energy* **141**, 2013-2044, doi:https://doi.org/10.1016/j.energy.2017.11.128 (2017).
3. Porteous, A. Why energy from waste incineration is an essential component of environmentally responsible waste management. *Waste management* **25**, 451-459 (2005).
4. Hasanbeigi, A. & Springer, C. California's Cement Industry: Failing the Climate Challenge. (Global Eciency Intelligence, San Francisco, CA, 2019).
5. Fernandez Pales, A. & Leung, Y. Technology Roadmap: Low-Carbon Transition in the Cement Industry. (International Energy Agency, 2018).
6. Chen, C. *et al.* A striking growth of CO2 emissions from the global cement industry driven by new facilities in emerging countries. *Environ. Res. Lett.* **17**, 044007, doi:10.1088/1748-9326/ac48b5 (2022).
7. Farfan, J., Fasihi, M. & Breyer, C. Trends in the global cement industry and opportunities for long-term sustainable CCU potential for Power-to-X. *Journal of Cleaner Production* **217**, 821-835, doi:https://doi.org/10.1016/j.jclepro.2019.01.226 (2019).
8. Janssens-Maenhout, G. *et al.* EDGAR v4. 3.2 Global Atlas of the three major greenhouse gas emissions for the period 1970–2012. *Earth System Science Data* **11**, 959-1002 (2019).
9. Le Quéré, C. *et al.* Global carbon budget 2016. *Earth System Science Data* **8**, 605-649 (2016).
10. Andrew, R. M. Global CO2 emissions from cement production, 1928–2018. *Earth Syst. Sci. Data* **11**, 1675-1710, doi:10.5194/essd-11-1675-2019 (2019).
11. Liu, Z. *et al.* Reduced carbon emission estimates from fossil fuel combustion and cement production in China. *Nature* **524**, 335-338 (2015).
12. Fernandez Pales, A. & Leung, Y. Technology Roadmap-Low-Carbon Transition in the

Cement Industry (International Energy Agency, 2018).

13 National Development and Reform Commission of China. Special plan for cement industry development. (China, 2006).

14 SCHORCHT, F., KOURTI, I., SCALET, B. M., ROUDIER, S. & SANCHO, L. D. Best Available Techniques (BAT) Reference Document for the Production of Cement, Lime and Magnesium Oxide: Industrial Emissions Directive 2010/75/EU:(Integrated Pollution Prevention and Control). (Luxembourg, 2013).

15 United States Environmental Protection Agency. Emission factors for greenhouse gas inventories. (US Environmental Protection Agency, US, 2021).

16 Dean, B., Dulac, J., Petrichenko, K. & Graham, P. Towards zero-emission efficient and resilient buildings: Global status report. (Paris, 2016).

17 International Energy Agency. Energy Technology Perspectives 2016. (IEA, Paris, 2016).

18 Rogelj, J. *et al.* Scenarios towards limiting global mean temperature increase below 1.5 C. *Nat. Clim. Chang.* **8**, 325-332 (2018).

19 Riahi, K. *et al.* The Shared Socioeconomic Pathways and their energy, land use, and greenhouse gas emissions implications: An overview. *Global Environmental Change* **42**, 153-168 (2017).

20 Gidden, M. J. *et al.* Global emissions pathways under different socioeconomic scenarios for use in CMIP6: a dataset of harmonized emissions trajectories through the end of the century. *Geoscientific Model Development Discussions*, 1-42 (2018).

21 International Energy Agency & the United Nations Environment Programme. 2018 Global Status Report: Towards a zero-emission, efficient and resilient buildings and construction sector. (2018).

22 Cao, Z., Shen, L., Løvik, A. N., Müller, D. B. & Liu, G. Elaborating the history of our cementing societies: an in-use stock perspective. *Environ. Sci. Technol.* **51**, 11468-11475 (2017).

23 Daigo, I., Iwata, K., Oguchi, M. & Goto, Y. Lifetime distribution of buildings decided by economic situation at demolition: D-based lifetime distribution. *Procedia CIRP* **61**, 146-151 (2017).

24 Zhou, W., Moncaster, A., Reiner, D. M. & Guthrie, P. Estimating Lifetimes and Stock Turnover Dynamics of Urban Residential Buildings in China. *Sustainability* **11**, 3720 (2019).

25 Zhou, W., O'Neill, E., Moncaster, A., Reiner, D. M. & Guthrie, P. Forecasting urban residential stock turnover dynamics using system dynamics and Bayesian model averaging. *Applied Energy* **275**, 115388 (2020).

26 Jain, S., Singhal, S. & Jain, N. K. Construction and demolition waste generation in cities in India: an integrated approach. *International Journal of Sustainable Engineering* **12**, 333-340 (2019).

27 Roussanaly, S. *et al.* Towards improved cost evaluation of Carbon Capture and Storage from industry. *International Journal of Greenhouse Gas Control* **106**, 103263, doi:https://doi.org/10.1016/j.ijggc.2021.103263 (2021).

28 D'Arcy, P. & Veroude, A. Housing trends in China and India. *Reserve Bank of Australia Bulletin*, 63-68 (2014).

29 Wang, Y., Höller, S., Viebahn, P. & Hao, Z. Integrated assessment of CO2 reduction technologies in China's cement industry. *International Journal of Greenhouse Gas Control*

20, 27-36, doi:https://doi.org/10.1016/j.ijggc.2013.10.004 (2014).

30 Garcia, M. & Berghout, N. Toward a common method of cost-review for carbon capture technologies in the industrial sector: cement and iron and steel plants. *International Journal of Greenhouse Gas Control* **87**, 142-158 (2019).

31 International Energy Agency. *Cement Technology Roadmap: Carbon Emissions Reductions up to 2050*. (2009).

32 ECRA (European Cement Research Academy) and CSI (Cement Sustainability Initiative). Development of State of the Art-Techniques in Cement Manufacturing: Trying to Look Ahead. (Duesseldorf, Geneva, 2017).

33 Hills, T., Leeson, D., Florin, N. & Fennell, P. Carbon Capture in the Cement Industry: Technologies, Progress, and Retrofitting. *Environ. Sci. Technol.* **50**, 368-377, doi:10.1021/acs.est.5b03508 (2016).

34 Obrist, M. D., Kannan, R., Schmidt, T. J. & Kober, T. Decarbonization pathways of the Swiss cement industry towards net zero emissions. *Journal of Cleaner Production* **288**, 125413, doi:https://doi.org/10.1016/j.jclepro.2020.125413 (2021).

35 Vatopoulos, K. & Tzimas, E. Assessment of CO2 capture technologies in cement manufacturing process. *Journal of Cleaner Production* **32**, 251-261, doi:https://doi.org/10.1016/j.jclepro.2012.03.013 (2012).

36 Cormos, A.-M. & Cormos, C.-C. Reducing the carbon footprint of cement industry by post-combustion CO2 capture: Techno-economic and environmental assessment of a CCS project in Romania. *Chemical Engineering Research and Design* **123**, 230-239, doi:https://doi.org/10.1016/j.cherd.2017.05.013 (2017).

37 Lisbona, P., Gori, R., Romeo, L. M. & Desideri, U. Techno-economic assessment of an industrial carbon capture hub sharing a cement rotary kiln as sorbent regenerator. *International Journal of Greenhouse Gas Control* **112**, 103524, doi:https://doi.org/10.1016/j.ijggc.2021.103524 (2021).

38 Atsonios, K., Grammelis, P., Antiohos, S. K., Nikolopoulos, N. & Kakaras, E. Integration of calcium looping technology in existing cement plant for CO2 capture: Process modeling and technical considerations. *Fuel* **153**, 210-223, doi:https://doi.org/10.1016/j.fuel.2015.02.084 (2015).

39 Xu, J.-H., Yi, B.-W. & Fan, Y. A bottom-up optimization model for long-term CO2 emissions reduction pathway in the cement industry: A case study of China. *International Journal of Greenhouse Gas Control* **44**, 199-216, doi:https://doi.org/10.1016/j.ijggc.2015.11.028 (2016).

40 Tomatis, M., Jeswani, H. K., Stamford, L. & Azapagic, A. Assessing the environmental sustainability of an emerging energy technology: Solar thermal calcination for cement production. *Science of The Total Environment* **742**, 140510, doi:https://doi.org/10.1016/j.scitotenv.2020.140510 (2020).

41 Xu, J.-H., Fleiter, T., Fan, Y. & Eichhammer, W. CO2 emissions reduction potential in China's cement industry compared to IEA's Cement Technology Roadmap up to 2050. *Applied Energy* **130**, 592-602, doi:https://doi.org/10.1016/j.apenergy.2014.03.004 (2014).

42 van Ruijven, B. J. *et al.* Long-term model-based projections of energy use and CO2 emissions from the global steel and cement industries. *Resources, Conservation and Recycling* **112**, 15-36, doi:https://doi.org/10.1016/j.resconrec.2016.04.016 (2016).

43 WBCSD Cement Sustainability Initiative (CSI) and European Cement Research Academy (ECRA). *Cement CO2 and Energy Protocol version 3.1*, <https://gccassociation.org/sustainability-innovation/gnr-gcca-in-numbers/> (2020).

44 World Bank. *GDP (current US$) - India*, <https://data.worldbank.org/indicator/NY.GDP.MKTP.CD?locations=IN> (2020).

45 Shan, Y. *et al.* Peak cement-related CO2 emissions and the changes in drivers in China. *Journal of Industrial Ecology* **23**, 959-971, doi:https://doi.org/10.1111/jiec.12839 (2019).

46 Intergovernmental Panel on Climate Change. *IPCC guidelines for national greenhouse gas inventories 2006*. Vol. 5 (Hayama, Japan: Institute for Global Environmental Strategies, 2006).

47 Miller, S. A. & Moore, F. C. Climate and health damages from global concrete production. *Nature Climate Change* **10**, 439-443, doi:10.1038/s41558-020-0733-0 (2020).

48 Guterres, A. World Economic Situation Prospects 2020. (UN Departments of Global Communications & Economic and Social Affairs, 2020).

49 Liu, J. *et al.* Carbon and air pollutant emissions from China's cement industry 1990–2015: trends, evolution of technologies, and drivers. *Atmospheric Chemistry Physics* **21**, 1627-1647 (2021).

50 Grubler, A. *et al.* A low energy demand scenario for meeting the 1.5 C target and sustainable development goals without negative emission technologies. *Nature energy* **3**, 515-527 (2018).

51 The European Cement Association. The role of cement in the 2050 low carbon economy. (The European Cement Association, Brussels, 2018).

52 Ali, M. B., Saidur, R. & Hossain, M. S. A review on emission analysis in cement industries. *Renewable and Sustainable Energy Reviews* **15**, 2252-2261, doi:https://doi.org/10.1016/j.rser.2011.02.014 (2011).

53 Hasanbeigi, A., Price, L. & Lin, E. Emerging energy-efficiency and CO2 emission-reduction technologies for cement and concrete production: A technical review. *Renewable Sustainable Energy Reviews* **16**, 6220-6238 (2012).

54 Benhelal, E., Zahedi, G., Shamsaei, E. & Bahadori, A. Global strategies and potentials to curb CO2 emissions in cement industry. *Journal of cleaner production* **51**, 142-161 (2013).

55 Ke, J., Zheng, N., Fridley, D., Price, L. & Zhou, N. Potential energy savings and CO2 emissions reduction of China's cement industry. *Energy Policy* **45**, 739-751, doi:https://doi.org/10.1016/j.enpol.2012.03.036 (2012).

56 Ishak, S. A. & Hashim, H. Low carbon measures for cement plant – a review. *Journal of Cleaner Production* **103**, 260-274, doi:https://doi.org/10.1016/j.jclepro.2014.11.003 (2015).

57 Georgiopoulou, M. & Lyberatos, G. Life cycle assessment of the use of alternative fuels in cement kilns: A case study. *Journal of Environmental Management* **216**, 224-234, doi:https://doi.org/10.1016/j.jenvman.2017.07.017 (2018).

58 Habert, G., Billard, C., Rossi, P., Chen, C. & Roussel, N. J. C. Cement production technology improvement compared to factor 4 objectives. *Cement Concrete Research* **40**, 820-826 (2010).

59 Environment UN, Scrivener, K. L., John, V. M. & Gartner, E. M. Eco-efficient cements: Potential economically viable solutions for a low-CO2 cement-based materials industry.

Cement Concrete Research **114**, 2-26 (2018).

60 Sharma, M., Bishnoi, S., Martirena, F. & Scrivener, K. Limestone calcined clay cement and concrete: A state-of-the-art review. *Cement and Concrete Research* **149**, 106564, doi:https://doi.org/10.1016/j.cemconres.2021.106564 (2021).

61 Dhandapani, Y., Sakthivel, T., Santhanam, M., Gettu, R. & Pillai, R. G. Mechanical properties and durability performance of concretes with Limestone Calcined Clay Cement (LC3). *Cement and Concrete Research* **107**, 136-151, doi:https://doi.org/10.1016/j.cemconres.2018.02.005 (2018).

62 Fennell, P. S., Davis, S. J. & Mohammed, A. Decarbonizing cement production. *Joule* **5**, 1305-1311, doi:https://doi.org/10.1016/j.joule.2021.04.011 (2021).

63 Miller, S. A., John, V. M., Pacca, S. A. & Horvath, A. Carbon dioxide reduction potential in the global cement industry by 2050. *Cement and Concrete Research* **114**, 115-124, doi:https://doi.org/10.1016/j.cemconres.2017.08.026 (2018).

64 Miller, S. A. Supplementary cementitious materials to mitigate greenhouse gas emissions from concrete: can there be too much of a good thing? *Journal of Cleaner Production* **178**, 587-598, doi:https://doi.org/10.1016/j.jclepro.2018.01.008 (2018).

65 Lothenbach, B., Scrivener, K. & Hooton, R. D. Supplementary cementitious materials. *Cement and Concrete Research* **41**, 1244-1256, doi:https://doi.org/10.1016/j.cemconres.2010.12.001 (2011).

66 Cancio Díaz, Y. *et al.* Limestone calcined clay cement as a low-carbon solution to meet expanding cement demand in emerging economies. *Development Engineering* **2**, 82-91, doi:https://doi.org/10.1016/j.deveng.2017.06.001 (2017).

67 IEAGHG. Deployment of CCS in the cement industry. (2013).

68 International Energy Agency. *Energy technology perspectives 2012: pathways to a clean energy system*. (OECD Publishing, 2012).

69 Vega, F. *et al.* Current status of CO2 chemical absorption research applied to CCS: Towards full deployment at industrial scale. *Applied Energy* **260**, 114313, doi:https://doi.org/10.1016/j.apenergy.2019.114313 (2020).

70 Carrasco-Maldonado, F. *et al.* Oxy-fuel combustion technology for cement production – State of the art research and technology development. *International Journal of Greenhouse Gas Control* **45**, 189-199, doi:https://doi.org/10.1016/j.ijggc.2015.12.014 (2016).

71 Cavalett, O., Cherubini, F. & Olsson, O. Deployment of bio-CCS in the cement sector: an overview of technology options and policy tools. (IEA Bioenergy, 2021).

72 Zhou, W. *et al.* Capturing CO2 from cement plants: A priority for reducing CO2 emissions in China. *Energy* **106**, 464-474, doi:https://doi.org/10.1016/j.energy.2016.03.090 (2016).

73 Guo, Y. *et al.* A review of low-carbon technologies and projects for the global cement industry. *Journal of Environmental Sciences* **136**, 682-697, doi:https://doi.org/10.1016/j.jes.2023.01.021 (2024).

74 Voldsund, M. *et al.* Comparison of Technologies for CO2 Capture from Cement Production—Part 1: Technical Evaluation. *Energies* **12** (2019).

75 Diego, M. E., Arias, B. & Abanades, J. C. Analysis of a double calcium loop process configuration for CO2 capture in cement plants. *Journal of Cleaner Production* **117**, 110-121, doi:https://doi.org/10.1016/j.jclepro.2016.01.027 (2016).

76 IPCC. IPCC Special Report on Carbon Dioxide Capture and Storage. (United Kingdom and New York, 2005).

77 Shi, F. *et al.* Toward a Low Carbon–Dematerialization Society: Measuring the Materials Demand and CO2 Emissions of Building and Transport Infrastructure Construction in China. *Journal of Industrial Ecology* **16**, 493-505 (2012).

78 Cao, Z. *et al.* Estimating the in-use cement stock in China: 1920–2013. *Resources, Conservation and Recycling* **122**, 21-31, doi:https://doi.org/10.1016/j.resconrec.2017.01.021 (2017).

79 Poponi, D. *et al. Energy technology perspectives 2016: towards sustainable urban energy systems*. (International Energy Agency, 2016).

80 Chatterjee, S., Kiss, B., Ürge-Vorsatz, D. & Teske, S. in *Achieving the Paris Climate Agreement Goals : Part 2: Science-based Target Setting for the Finance industry — Net-Zero Sectoral 1.5℃ Pathways for Real Economy Sectors* (eds Sven Chatterjee Teske, Souran, Benedek Kiss, Diana Ürge-Vorsatz, & Sven Teske) 161-185 (Springer International Publishing, 2022).

81 Hong, L., Zhou, N., Fridley, D., Feng, W. & Khanna, N. in *ACEEE summer study on energy efficiency in buildings.* (eds Lixuan Hong *et al.*) 146-157.

82 B. Müller, D. Stock dynamics for forecasting material flows—Case study for housing in The Netherlands. *Ecological Economics* **59**, 142-156, doi:https://doi.org/10.1016/j.ecolecon.2005.09.025 (2006).

83 Bergsdal, H., Brattebø, H., Bohne, R. A. & Müller, D. B. Dynamic material flow analysis for Norway's dwelling stock. *Building Research Information* **35**, 557-570 (2007).

84 Müller, E., Hilty, L. M., Widmer, R., Schluep, M. & Faulstich, M. Modeling metal stocks and flows: a review of dynamic material flow analysis methods. *Environmental science technology* **48**, 2102-2113 (2014).

85 Pauliuk, S. & Müller, D. B. The role of in-use stocks in the social metabolism and in climate change mitigation. *Global Environmental Change* **24**, 132-142, doi:https://doi.org/10.1016/j.gloenvcha.2013.11.006 (2014).

86 Lauinger, D., Billy, R. G., Vásquez, F. & Müller, D. B. A general framework for stock dynamics of populations and built and natural environments. *Journal of Industrial Ecology* **25**, 1136-1146 (2021).

87 Berrill, P. & Hertwich, E. Material flows and GHG emissions from housing stock evolution in US counties, 2020–60. *Buildings and Cities* **2**, 599-617, doi:10.5334/bc.126 (2021).

88 Schiller, G. Urban infrastructure: challenges for resource efficiency in the building stock. *Building Research & Information* **35**, 399-411, doi:10.1080/09613210701217171 (2007).

89 Yang, W. *Modeling the evolution of the Chinese building stock in a sustainable perspective (in Chinese)* Ph.D. thesis, Tianjin University, (2006).

90 Ellis, J. *An Initial View on Methodologies for Emission Baselines: Cement Case Study, OECD and IEA Information Paper.* (Organization for Economic Cooperation Development, 2000).

91 Chen, Z. *et al.* Assessment of regional greenhouse gas emission from beef cattle production: A case study of Saskatchewan in Canada. *Journal of Environmental Management* **264**, 110443, doi:https://doi.org/10.1016/j.jenvman.2020.110443 (2020).

92 Sohani, A., Rezapour, S. & Sayyaadi, H. Comprehensive performance evaluation and

demands' sensitivity analysis of different optimum sizing strategies for a combined cooling, heating, and power system. *Journal of Cleaner Production* **279**, 123225, doi:https://doi.org/10.1016/j.jclepro.2020.123225 (2021).

93 Brown, L. *et al.* An inventory of nitrous oxide emissions from agriculture in the UK using the IPCC methodology: emission estimate, uncertainty and sensitivity analysis. *Atmospheric Environment* **35**, 1439-1449, doi:https://doi.org/10.1016/S1352-2310(00)00361-7 (2001).

94 Turgut, S. S., Feyissa, A. H., Küçüköner, E. & Karacabey, E. Uncertainty and sensitivity analysis by Monte Carlo simulation: Recovery of trans-resveratrol from grape cane by pressurised low polarity water system. *Journal of Food Engineering* **292**, 110366, doi:https://doi.org/10.1016/j.jfoodeng.2020.110366 (2021).
